# Computational Characterizing Necroptosis Reveals Implications for Immune Infiltration and Immunotherapy of Hepatocellular Carcinoma

**DOI:** 10.3389/fonc.2022.933210

**Published:** 2022-07-07

**Authors:** Jun Zhu, Tenghui Han, Shoujie Zhao, Yejing Zhu, Shouzheng Ma, Fenghua Xu, Tingting Bai, Yuxin Tang, Yungang Xu, Lei Liu

**Affiliations:** ^1^ Department of Gastroenterology, Daping Hospital, Army Medical University, Chongqing, China; ^2^ Department of General Surgery, The Southern Theater Air Force Hospital, Guangzhou, China; ^3^ Department of Neurology, Xijing Hospital, Fourth Military Medical University, Xi’an, China; ^4^ Department of General Surgery, Tangdu Hospital, Fourth Military Medical University, Xi’an, China; ^5^ Department of Surgery, Tangdu Hospital, Fourth Military Medical University, Xi’an, China; ^6^ Department of Cell Biology and Genetics, School of Basic Medical Sciences, Xi’an Jiaotong University Health Science Center, Xi’an, China; ^7^ Centre for Computational Systems Medicine, School of Biomedical Informatics, The University of Texas Health Science Centre at Houston, Houston, TX, United States

**Keywords:** necroptosis, tumor microenvironment, immunotherapy, chemotherapy, tumor-infiltrating cells, hepatocellular carcinoma

## Abstract

Necroptosis is a programmed form of necrotic cell death in regulating cancer ontogenesis, progression, and tumor microenvironment (TME) and could drive tumor-infiltrating cells to release pro-inflammatory cytokines, incurring strong immune responses. Nowadays, there are few identified biomarkers applied in clinical immunotherapy, and it is increasingly recognized that high levels of tumor necroptosis could enhance the response to immunotherapy. However, comprehensive characterization of necroptosis associated with TME and immunotherapy in Hepatocellular carcinoma (HCC) remains unexplored. Here, we computationally characterized necroptosis landscape in HCC samples from TCGA and ICGA cohorts and stratified them into two necroptosis clusters (A or B) with significantly different characteristics in clinical prognosis, immune cell function, and TME-landscapes. Additionally, to further evaluate the necroptosis levels of each sample, we established a novel necroptosis-related gene score (NRGscore). We further investigated the TME, tumor mutational burden (TMB), clinical response to immunotherapy, and chemotherapeutic drug sensitivity of HCC subgroups stratified by the necroptosis landscapes. The NRGscore is robust and highly predictive of HCC clinical outcomes. Further analysis indicated that the high NRGscore group resembles the immune-inflamed phenotype while the low score group is analogous to the immune-exclusion or metabolism phenotype. Additionally, the high NRGscore group is more sensitive to immune checkpoint blockade-based immunotherapy, which was further validated using an external HCC cohort, metastatic melanoma cohort, and advanced urothelial cancer cohort. Besides, the NRGscore was demonstrated as a potential biomarker for chemotherapy, wherein the high NRGscore patients with more tumor stem cell composition could be more sensitive to Cisplatin, Doxorubicin, Paclitaxel-based chemotherapy, and Sorafenib therapy. Collectively, a comprehensive characterization of the necroptosis in HCC suggested its implications for predicting immune infiltration and response to immunotherapy of HCC, providing promising strategies for treatment.

## Introduction

Hepatocellular carcinoma (HCC) is acknowledged to be one of the most common malignant tumors globally, accounting for one-third of cancer mortalities (1). Risk factors of HCC progression contain metabolic disorders, viral infections by hepatitis B virus and hepatitis C virus, absorption of the aflatoxin-contaminated meal, and cirrhosis out of alcoholic hepatitis (2–4). Though early examination and intervention have achieved success, the ratio of diagnosis at the early stage remains low (5). The majority of HCC patients are diagnosed at an advanced stage (6). Although plentiful efforts were made in both diagnosis and treatment of HCC, its overall survival (OS) rate remains unfavorable. Seventy percent of HCC patients have recurrent neoplasm 5 years after resection (7). Consequently, the 5-year survival rate of HCC patients turns out unsatisfactory (8).

Chemotherapy failure has been a major obstacle during cancer treatment, among which apoptosis resistance (innate or acquired) is widely accepted mechanistically. How to bypass the apoptosis pathway and induce effective cell death pathways are becoming crucial in the treatments of HCC. Like apoptosis and necrosis, necroptosis is a novel programmed form of necrotic cell death that is mainly manipulated by receptor-interacting Protein Kinase 1 (RIPK1), RIPK3, and Mixed Lineage Kinase domain-like (MLKL) (9). Accumulating data demonstrate that necroptosis plays a crucial role in the regulation of cancer biology, including cancer progression (10, 11), cancer metastasis (12), cancer Immunosurveillance (13), and cancer subtypes (14, 15).

With the advent of immunotherapy, such as immune checkpoint blockades (ICBs), only a few HCC patients have been reported to gain clinical benefits from ICBs (16, 17). To date, Some predictors of response to ICBs were identified, including PD-L1 expression (18), the degree of cytotoxic T cell infiltration (19), tumor mutational burden (TMB) (20), mismatch repair deficiency (21), and activated Wnt/β-catenin signaling (22), et al. However, to our best knowledge, these above biomarkers have been confirmed in HCC (23). There is a rationale supporting the development of some novel biomarkers for ICBs in HCC patients. Recently, a few studies revealed crosstalk between necroptosis and antitumor immunity (24). Necroptosis not only has direct interaction with immune cells like dendritic cells (DCs) and natural killer T cells (NKT) but also could initiate an adaptive immune system by promoting DC cells and macrophages to release pro-inflammatory cytokines into the tumor microenvironment (TME), incurring strong immune responses (25). In *in vivo* and *in vitro* experiments, necroptotic tumor cells were shown to induce antitumor immunogenicity through the cross-priming and proliferation of CD8^+^ T cells (26). Furthermore, with the advance in nanomedicine, necroptotic cancer cells could boost antitumor immunity by administrating cell-mimicry nanovaccine with a tailored immunostimulatory modality (26). In addition, the effect of tumor regression by nanovaccine could be enhanced by combination with ICBs (26). However, the discovery of a specific necroptosis marker, a thorough investigation of the molecular mechanism, and a clarification of its crosstalk with other cell death machinery and its interaction with the immune system should be urgently further investigated. Taken together, necroptosis has a close relationship with TME and antitumor immunity, suggesting necroptosis could become a novel biomarker for chemotherapy and immunotherapy of HCC. However, it’s rarely depicted for necroptosis characteristics from a multi-omics perspective and its correlation with ICBs and chemotherapy in HCC.

In this study, we uncovered that necroptosis regulators were clinically predictive and independent factors for HCC patients by survival analysis and unsupervised clustering analysis. Necroptosis regulators could distinguish patients into two necroptosis clusters (Nclusters A and B) and different Nclusters were enriched into several TME-related pathways and various immune infiltration pathways. Therefore, we proposed a hypothesis: necroptosis could become a new and indispensable factor for HCC prognosis and TME characterization. A novel necroptosis-related gene score (NRGscore) was established by the Lasso algorithm and multiple Cox regression analysis. In TME cell infiltration of HCC, NRGscore had a negative relation with activated NK cells and macrophage M1 cells while positively associated with Treg cells. NRGscore had meaningful guidance for patients, where the high NRGscore group could be more sensitive to ICB treatment, which was validated in other independent cancer cohorts, as well. As for chemotherapy and targeted therapy, due to more stem cells and enhanced cell proliferation, the high NRGscore group could be more sensitive to Cisplatin, Doxorubicin, Paclitaxel-based chemotherapy, and Sorafenib targeted therapy than its counterparts. Taken together, the prognostic NRGscore system could help to dissect the TME characterization of HCC and to interpret the clinical responses to chemotherapies and immunotherapies, providing new target molecules for the treatment of cancers.

## Methods

### Data Sources

The workflow of this study is shown in **Figure S1**. Necroptosis-related genes were collected based on the GESA necroptosis gene set and published literature **(** **Table S1** **)**. Clinical information and mRNA expression matrixes of HCC patients were acquired from The Cancer Genome Atlas (TCGA, https://portal.gdc.cancer.gov/ ) database and International Cancer Genome Consortium (ICGC, https://dcc.icgc.org/) database. Then we transformed FPKM values into transcripts per kilobase million (TPM) values. The basic clinical information of HCC datasets in the study is summarized in **Table S2**. The somatic mutation data of the TCGA and ICGC cohort were downloaded from the UCSC Xena (https://gdc.xenahubs.net/). Additionally, the TCGA cohort was applied to Copy Number Variation (CNV) analysis. Another HCC validated cohort (GSE54236) (27, 28) and corresponding clinical features were collected to assess predictive power in our study.

### Identification of Significant Mutational Genes

The “maftools” R package was applied to process the mutation annotation format (maf) data, and the “MutSigCV” algorithm was implemented to screen the significant mutational genes (SMGs) (29). The significance of nonsilent somatic mutations in a gene was measured based on the background mutation rates by silent mutation. The false discovery rates (FDR (30)) were then calculated, and genes with statistical significance (FDR ≤ 0.1) were set as SMGs. Then, waterfall plots were employed to visualize the mutation information of these significant SMGs in the TCGA cohort. Besides, Fisher’s test was applied to detect the mutually exclusive or co-occurring ratio of necroptosis-related genes. By adopting the “ExtractSignatures” function that applies the Bayesian nonnegative matrix factorization-based framework, we determined the mutational signatures using the genomic data. The optimal number of mutational signatures for the TCGA cohort could be detected by the “SignatureEnrichment” function and then it automatically assigned a given signature to each sample.

### Unsupervised Cluster Analysis

Based on the expression of necroptosis-related genes, unsupervised clustering analysis was performed to stratify patients into different clusters. The “ConsensuClusterPlus” R package was adopted to determine the number of clusters and guarantee the stability of classification (31). The principal component analysis (PCA) was utilized to investigate gene-expression arrays among distinct clusters.

### Tumor Infiltration Cell and Immune-Related Function Analysis

To estimate the abundance and activity of tumor infiltration cells (TICs) in HCC, the single-sample gene set enrichment analysis (ssGSEA) algorithm was implemented by using the “GSVA” R package (32). In addition, CIBERSORT (33), an analysis algorithm based on the immune gene set, was also used to evaluate the TICs levels of HCC. The algorithm was run for 1000 permutations and HCC samples with an output P < 0.05 were selected as previously reported (34, 35). Twenty-three types of TICs were comprised of adaptive immune cells (B cells, T cells, CD8 T cells, T follicular helper (Tfh), Th1, Th2, Th17, and Treg cells), and innate immune cells (NK cells, CD56 dim NK cells, CD56 bright NK cells, DCs, plasmacytoid DCs, immature DCs, neutrophils, mast cells, and macrophages). Besides, immune-related pathways (such as cytolytic activity, T-cell costimulation, inflammation-promoting, and para-inflammation) were also calculated *via* ssGSEA. The biosimilarity of infiltrating immune cells and immune-related functions were estimated by the Gaussian fitting model.

### Functional Annotation Analysis

To investigate the enrichments of biological processes, cell components, and molecular function pathways, Gene Ontology (GO) analysis, and Kyoto Encyclopedia of Genes and Genomes (KEGG) signaling pathway analysis were conducted using the “clusterProfiler” R package. Additionally, Gene Set Enrichment Analysis (GSEA) (36) was adopted to identify the differences in DEGs between the distinct clusters in the enrichment of the KEGG pathway. Permutations were performed 1000 times for each analysis. P-value < 0.05 and adjusted P-value (Q value) < 0.05 were considered statistically significant.

### Differentially Expressed Genes Analysis

To identify necroptosis-related genes, patients were classified into distinct groups according to sample types, necroptosis clusters, and NRGscore, respectively. The “limma” R package was utilized to determine DEGs between different groups (37). The significance filtering criteria for determining DEGs were set as adjusted P-value < 0.001 and fold change > 1.5.

### Dimension Reduction and Construction of NRGscore

Prognostic genes were identified from DEGs by performing the univariate Cox regression. The least absolute shrinkage and selection operator (Lasso) regression was conducted to necroptosis gene signature *via* utilizing the “glmnet” R package. Responding coefficients (β) of the signature were verified. Additionally, the signature was calculated by the following equation: NRGscore = ∑(*exp*(gene)∗β), where *exp* indicated RNA expression of HCC samples.

### Clinical Characteristic Evaluation of NRGscore

The survival curves for the different subgroups were generated by Kaplan-Meier (K-M) methods. The areas under the curve (AUC) of the receiver operating characteristic (ROC) curve were applied to assess the predictive value of gene signature. The nomogram was built based on the NRGscore and clinicopathologic characteristics including age, gender, T stage, and N stage to predict the survival probability of 1-, 3-, and 5-year OS of HCC patients. The calibration curve of the nomogram was plotted to estimate the prediction possibilities according to the observed survival rates. The nomogram and calibration plots were generated based on the “rms” R package.

### Estimation of Tumor Immune Microenvironment and Tumor Mutational Burden

To further dissect the immune landscape of HCC, the “ESTIMATE” package in R was used to evaluate the immune, stromal, and ESTIMATE scores, which reflect the ratio of the immune/stromal components of the tumor immune microenvironment (TME). To determine the tumor mutational burden (TMB) of each patient, we also counted the nonsynonymous and synonymous mutation counts in the TCGA cohort.

### Assessment of Clinical Response to the Immunotherapy

TIDE (38) algorithm and immunophenoscore (IPS) function (39) were prevalently recognized to be effective methods to predict cancer patients’ response to immunotherapy. There are two main mechanisms to immune escape and resistance to ICB-based immunotherapy (40, 41): (1) high levels of dysfunctional CTL; (2) immunosuppressive factors to exclude T cells from the tumor region. TIDE algorithm integrates these two immune-escape mechanisms and was used to predict patients’ response to ICB-based therapy based on the transcriptome. According to characterizing the determinant factors of cancer immunogenicity and antigenomes, we stratified HCC patients into different IPS groups. HCC sample is more immunogenic when the z-score of IPS is higher (42). Two immunotherapy cohorts, metastatic melanoma cohort treated with Nivolumab (19), and advanced urothelial cancer cohort with the intervention of atezolizumab (IMvigor210, http://research-pub.gene.com/IMvigor210CoreBiologies/) (43) were downloaded to evaluate the predictive value of the necroptosis score system for immunotherapy. In addition, the TIDE website (http://tide.dfci.harvard.edu/login/) was used to further evaluate the predictive power of other cancer cohorts by inputting gene coefficients. The raw gene expression data of all cohorts were normalized according to previous literature (44, 45).

### Dissection of Cancer Stem Cell And Prediction of Chemotherapeutic Drugs Sensitivity

To evaluate the cancer stem cell ratio and differentiation degree of HCC, the “limma” and “corrplot” R packages were applied. We adopted Spearman’s method to explore the correlation between NRGscore and cancer stem cells. In addition, based on the information retrieved from the Genomics of Drug Sensitivity in Cancer database (https://www.cancerrxgene.org/ ), we estimate the sensitivity of different chemotherapeutic drugs between high and low NRGscore subgroups. The prediction process used was the “pRRophetic” package (46) where the half-maximal inhibitory concentration was calculated by ridge regression model based on gene expression profiles.

### Statistical Analysis

All statistical analyses were conducted by R software (http://www.R-project.org, version 4.1.2), PERL programming language (version 5.32.1.1, https://www.perl.org/). K-M curve analysis with a log-rank test was utilized to compare OS between diverse subgroups. Mann-Whitney and Kruskal-Wallis test with adjusted P-values were employed to compare either ssGSEA scores of immune cells or functions of the distinct clusters as indicated in the article. P-value < 0.05 was statistically significant.

## Results

### Hepatocellular Carcinoma Was Well Characterized by Necroptosis Genes

**Figure 1:**
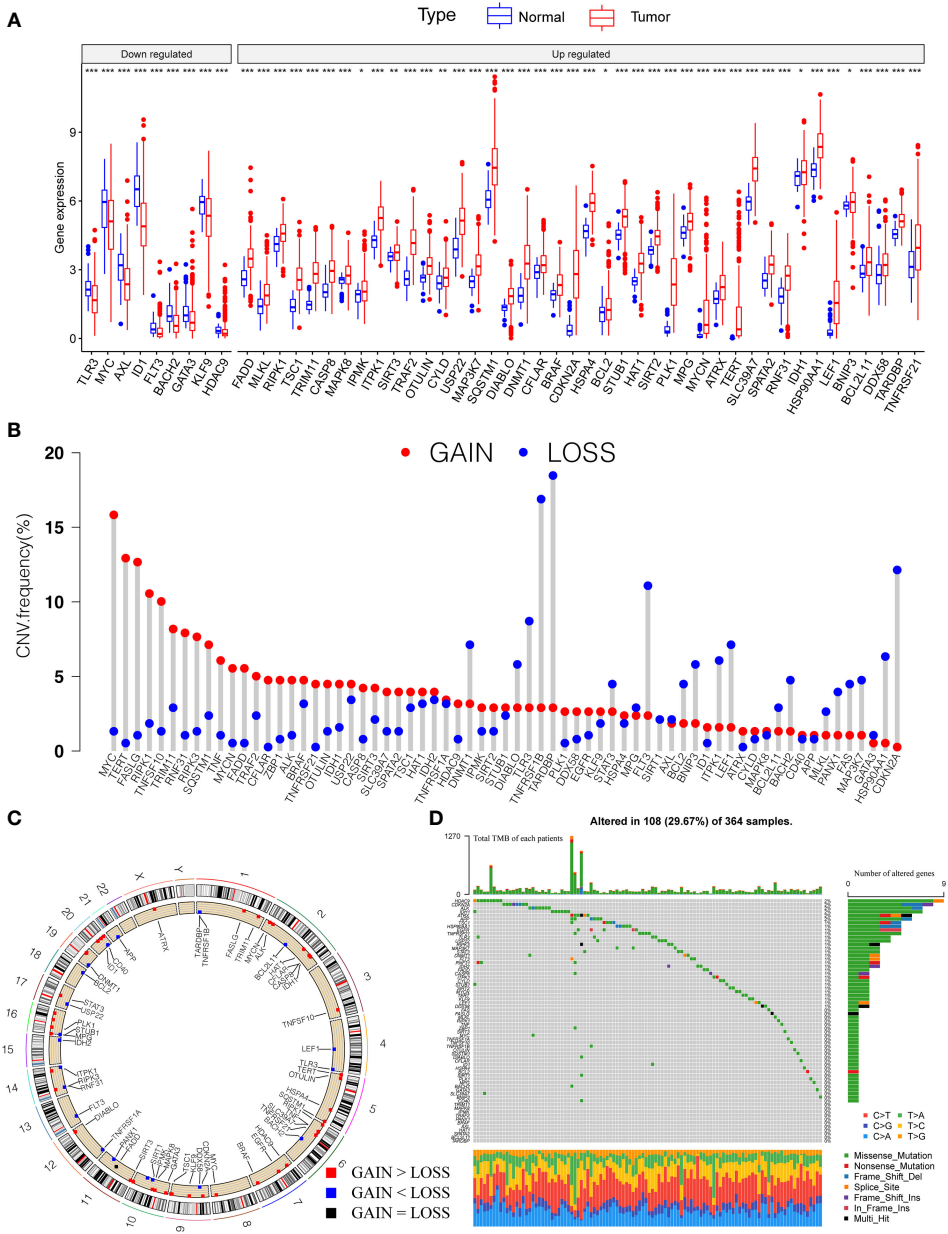
Genetic landscape and expression variation of necroptosis regulators in HCC. **(A)** The differential expression level of necroptosis regulators between tumor and normal tissues. **(B)** CNV alternation of necroptosis regulators in tumor tissues. Column represented the frequency of the variations. The green dot represented the deletion of CNV. The red dot represented the amplification of CNV. **(C)** Location of CNV of necroptosis regulators in chromosomes. Red dots represent genes gain than loss, blue dot presents genes loss than gain and the black dot means loss equal gain. **(D)** One hundred eight patients (29.67%) exhibited various genetic alterations, including missense, nonsense, splice, frameshift, and multiple mutations. Each column indicated individual HCC patients, and the upper bar diagram exhibited the TMB of HCC patients. The right number represented the mutation frequency, and the bar diagram on the right exhibited the proportion of each genetic alteration. P < 0.05 *; P < 0.01**; P < 0.001***. HCC, hepatocellular carcinoma; CNV, copy number variation; TMB, tumor mutation burden.

Firstly, we investigated 67 necroptosis genes (**Table S1**) regarding their expression and genetic variations between tumor and paratumor samples in TCGA cohorts. Fifty-one necroptosis genes presented significantly differential expression, of which 42 genes were upregulated and nine genes were downregulated in tumors (**Figure 1A**). Many differentially expressed necroptosis genes have been reported to be involved in modifying the microenvironment of HCC (15), including upregulated SIRT1/2 (47) and RIPK1 (48). It is well known that genetic variations, such as copy number variations (CNV) and mutations, result in perturbations of gene expression during tumorigenesis of HCC. The investigation of CNV alternation frequency showed a prevalent change in 67 necroptosis genes, and we observed that 44 genes exhibited the amplification in copy numbers and 23 genes showed depletion of CNVs (**Figures 1B, C**). Additionally, a total of 108 of 364 (29.67%) patients exhibited at least one type of mutation (**Figure 1D**). The most highly mutated genes, such as MYC, TERT, FASLG, RIPK1, TNFSF10, TARDBP, and CDKN2A, have been well characterized in liver cancer (15, 48), diffuse large B-cell lymphoma (49), neuroblastoma (50), and kidney renal clear cell carcinoma (51, 52). Especially, as the three most important necroptosis-driving molecules (53), RIPK1 and MLKL were upregulated in tumors and CNV of RIPK1 revealed amplification more than depletion, while RIPK3 presented no significant alternation in genomic expression. Extrapolating from all the above results, necroptosis genes present high heterogeneity among RNA expression, CNV, and mutations in HCC samples, which shows promise in tumorigenesis and development in HCC.

### Necroptosis Clusters Exhibit Distinct Prognosis, Biological Functions, and Immune Characteristics

**Figure 2:**
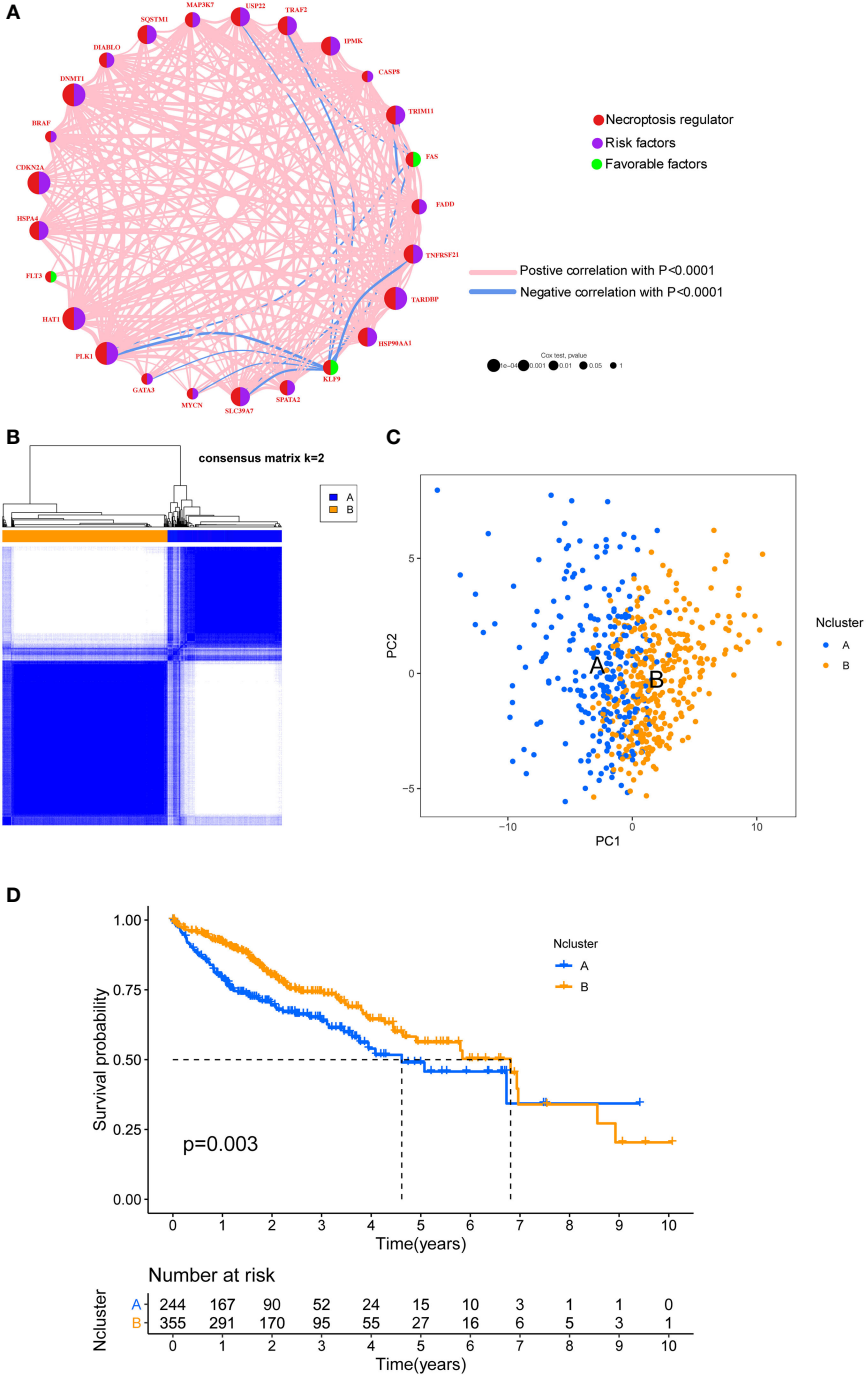
Unsupervised clustering of necroptosis regulators. **(A)** Interaction network of necroptosis regulators in HCC. Necroptosis regulators were indicated by red circles. Risk factors were indicated by purple circles, and favorable factors were indicated by green circles. Different sizes of the circle represented the different P-values. Red lines showed the positive correlation, and blue lines showed the negative correlation. **(B)** Consensus clustering matrix for k =2. **(C)** The feature distribution between different two Nclusters was plotted *via* PCA. **(D)** Kaplan-Meier curves of the OS for the two Nclusters of HCC patients. Ncluster, necroptosis cluster; PCA, principal component analysis; OS, overall survival; HCC, hepatocellular carcinoma.

To integrate the prognostic value of these necroptosis genes, we sought to construct a prognosis and correlation network according to the clinical outcomes of HCC patients (**Figure 2A**). Three necroptosis genes were favorable factors while 22 genes were risk factors, which disclosed that necroptosis may play a tumor-promoting role in HCC. Interestingly, we found that a positive correlation between prognostic necroptotic genes occurred more frequently than negative connections. To further explore the clinical role of necroptosis genes in HCC, we collected 599 clinical samples from TCGA and ICGC datasets and the basic clinical parameters are shown in **Table S2**. Based on expression profiles of necroptosis genes, we implemented unsupervised clustering to analyze the HCC samples from two cohorts and classified patients into qualitatively different subgroups. Two distinct subgroups were ultimately identified, including 244 cases in subgroup A and 355 cases in subgroup B, which were referred to as necroptosis clusters (Ncluster) A and B, respectively (**Figure 2B**). Additionally, PCA results showed necroptosis genes could vividly distinguish one subgroup from another (**Figure 2C**). K-M curves for the two subgroups revealed the prominent survival advantage in Ncluster B compared to A (**Figure 2D**). Moreover, correlations between two clusters and other clinicopathological parameters, including age, gender, TNM stage, tumor stage, and tumor grade, are shown in the heatmap (**Figure S2**). Ncluster A was characterized by higher levels of necroptosis and more advanced clinical stage and grade, thus resulting in a poor prognosis.

**Figure 3:**
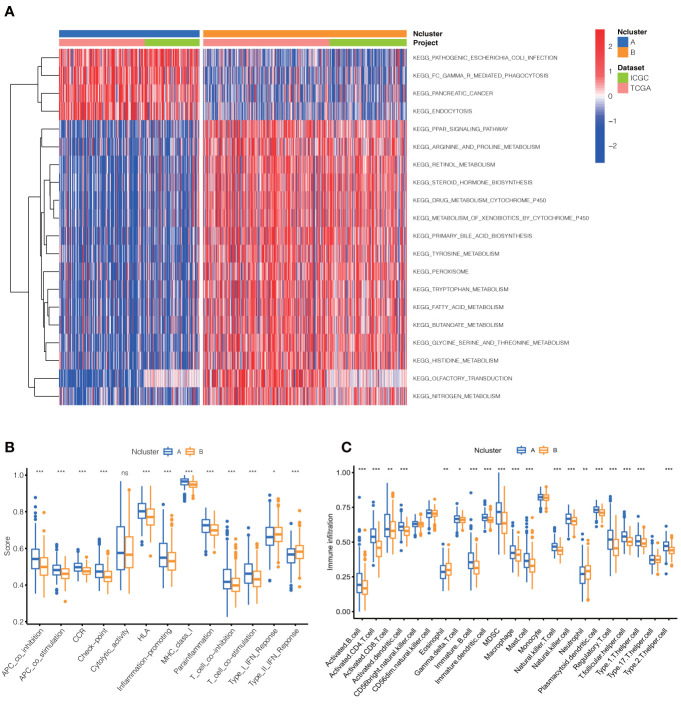
Dramatic difference of biological features between Ncluster A and B. **(A)** Heatmap showed various KEGG pathways were enriched in Ncluster A and B. **(B)** Immune function and infiltrating immune cells **(C)** altered in two Nclusters shown by violin diagram. The heatmap was used to visualize these biological processes, and red represented activated pathways and blue represented inhibited pathways. Ncluster, necroptosis cluster.

To further explore the potential biological behavior of necroptotic patterns, we performed GSVA analysis between two Nclusters. In KEGG signal pathways, Ncluster B was characterized by enhanced metabolism pathways, such as drugs, steroid hormone, bile acid, and fatty acid metabolism, while Ncluster A was characterized by upregulated cell endocytosis and phagocytosis pathways (**Figure 3A**). The tumor-infiltrating cells and immune functions play a critical role in the process of tumorigenesis and migration in HCC (54). In immune pathways of GSVA, we found Ncluster A possess more pathways for activated immune cell than Ncluster B (**Figure S3A**), suggesting N cluster A had a trend to immune-inflamed phenotype and Ncluster B was inclined to immune-excluded phenotype. Next, we investigated the difference between TICs and immune-related functions in two clusters by ssGSEA analysis. As vividly shown in **Figure 3B**, 10 immune functions were markedly downregulated and only IFN-response function upregulated in Ncluster B, which revealed that Ncluster B had less complicated TME. Consistent with results of immune pathways, there was significantly different TIC abundance in two subgroups, among which 19/23 TICs varied immensely, including 17 highly expressed types and only two downregulated kinds of TICs in Ncluster A (**Figure 3C**). In addition, we also determined 2988 Ncluster-related DEGs (**Table S3**) by limma R packages and conducted GO and KEGG enrichment analysis based on the Ncluster DEGs. Likely, DEGs were enriched in cell adhesion (EMT pathways) and T cell activation pathways (**Figure S3B**), PI3K-AKT pathways, and abundant immune cell regulation pathways (**Figure S3C**). Collectively, necroptosis could classify patients into two distinct subgroups and Ncluster A tends to have immune-inflamed phenotype and complex tumor-infiltrating patterns while Ncluster B was inclined to immune-excluded phenotype and metabolism phenotype.

### Necroptosis Patterns Define Necroptosis-Related Gene Clusters and NRGscore

**Figure 4:**
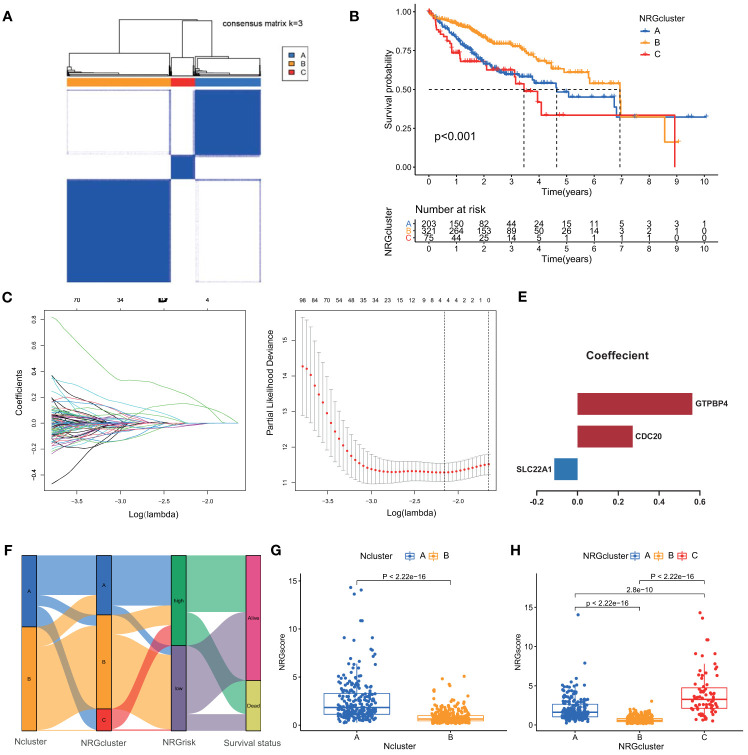
Identification of necroptosis genomic classification and construction of NRGscore. **(A)** Consensus clustering matrix for k =3. **(B)** Kaplan-Meier curves indicated necroptosis genomic phenotypes were markedly related to the OS of 599 patients in TCGA and ICGC cohorts, of which 203 cases were in gene cluster A, 321 cases in gene cluster B, and 75 cases in gene cluster C. **(C)** The partial likelihood deviance plot. **(D)** The Lasso regression coefficient profiles. **(E)** Coefficient of three core necroptosis-related DEGs. **(F)** The alluvial diagram exhibited the correlation of Ncluster, NRGcluster, NRGscore, and survival status. **(G)** Differences in NRGscore between two Nclusters in TCGA and ICGC cohorts. The upper and lower ends of the boxes represented the interquartile range of values. The lines in the boxes represented median value, and dots showed outliers. **(H)** Differences in NRGscore among three NRGclusters in TCGC and ICGC cohorts. P < 0.05 *; P < 0.01**; P < 0.001***. NRGscore, necroptosis-related gene score; OS, overall survival; TCGA, the cancer genome atlas; ICGC, international cancer genome consortium; Lasso, least absolute shrinkage and selection operator; DEGs, differentially expressed genes; Ncluster, necroptosis cluster; NRGclusters, necroptosis-related genes clusters.

To further evaluate the underlying biological role of necroptosis in clinical outcomes and tumor-infiltrating traits, we screened 2988 Ncluster related DEGs and determined 1819 prognostic genes by univariate Cox analysis (**Table S4**). Similarly, an unsupervised clustering method was used to accurately classify HCC patients into three stable gene clusters, which was termed as necroptosis-related gene cluster (NRGcluster), including 203 cases in NRGcluster A, 321 cases in NRGcluster B, and 75 cases in NRGcluster C (**Figure 4A**). The three NRGclusters had dramatic prognostic differences in HCC patients: NRGcluster B was proven to possess better prognostic outcomes, while patients in NRGcluster C were associated with poorer outcomes (**Figure 4B**). Furthermore, the three NRGclusters exhibited the different Nclusters, necroptosis gene expression (**Figure S4A**), among which NRGclusters had nearly Ncluster B subgroups and NRGcluster C was included in Ncluster A. Besides, the three NRGclusters had distinct expression profiles of necroptosis genes (**Figure S4A, B**). Among them, NRGcluster A or C had more expression of 51 than NRGcluster B while only six necroptosis regulators were highly expressed in Ncluster B compared to A or C, which revealed that the expression level of necroptosis genes had a negative relationship with HCC clinical prognosis and NRGcluster could represent necroptosis patterns of each HCC sample.

Integrating the evidence above, we found that necroptosis played an irreplaceable role in affecting prognosis, remodeling TME, and regulating immune reaction in HCC. However, the Ncluster or NRGcluster could not accurately clarify individual necroptosis patterns, and to further explore the heterogeneity and complexity of necroptosis, the Lasso algorithm was employed to quantify individual patients. Based on the optimal value of λ (λ=3), we constructed a necroptosis scoring system, termed as NRGscore (**Figures 4C, D**) by adopting multivariate COX regression analysis. Three hub genes were comprised, including GTPBP4 (Coefficient = 0.5632), CDC20 (Coefficient = 0.271), and SLC22A1 (Coefficient = -0.1136) (**Figure 4E**). The NRGscore was calculated by RNA expression multiplied by its corresponding coefficient.

The alluvial diagram visualized the quantification changes of patients and exhibited the interaction of Ncluster, NRGcluster, NRGscore, and survival state (**Figure 4F**). Results indicated that Ncluster A was mainly linked to a higher NRGscore, whereas Ncluster B exhibited a lower score (**Figure 4G**). Remarkably, NRGcluster C showed the highest NRGscore, followed by NRGcluster A, while NRGcluster B revealed the lowest scores (**Figure 4H**). Then, we investigated the relationship between NRGscore and necroptosis-driving regulators (RIPK1, RIPk2, and MLKL) and found NRGscore was positively linked to the expression of these three regulators (**Figure S5**). Additionally, we further stratified patients into high NRGscore and low NRGscore groups according to the median cut-off value derived from the R Survminer package. Next, we investigated the RNA expression levels of necroptosis genes, and the results exhibited that most (46/49) genes were highly expressed and only three (3/49) were downregulated in the high NRGscore subgroup (**Figure S6A**). Taken together, NRGcluster could accurately represent overall necroptosis levels and the NRGscore system could depict individual necroptosis patterns of HCC patients.

### NRGscore Is Highly Predictive of Clinical Outcomes for HCC

**Figure 5:**
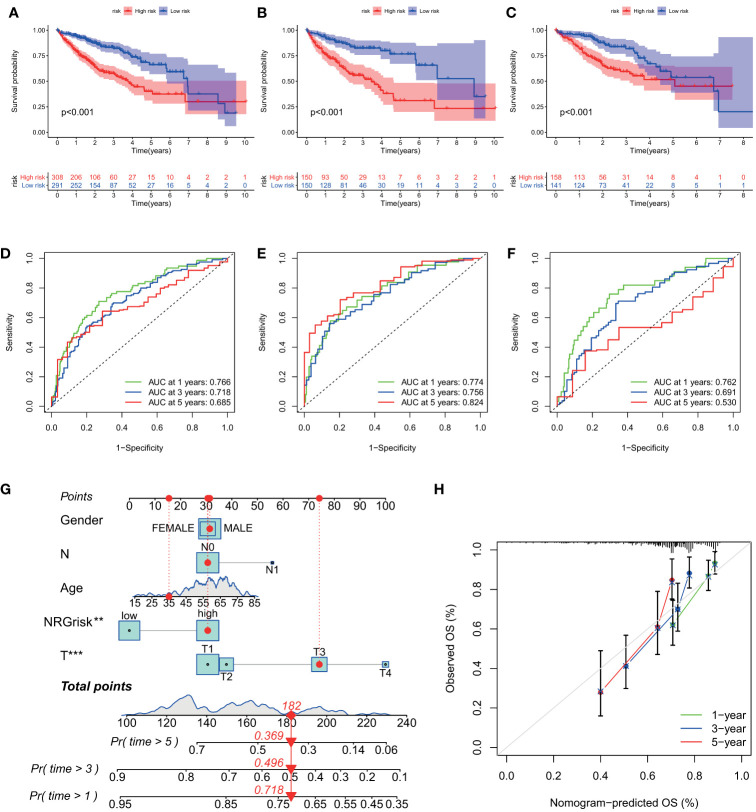
Evaluation of predictive power of NRGscore in training and test cohorts. **(A, C)** Kaplan-Meier curves showed that the high NRGrisk group had a more inferior OS than the low NRGrisk group in TCGA and ICGC cohorts, of which 599 cases were in the overall set **(A)**, 300 cases were in training set **(B)**, and 299 cases were in the test set **(C)**. **(D-F)** ROC of NRGscore scheme: Areas under the curve of 1-, 3-, and 5-year OS in the overall set **(D)**, training set **(E)**, and test set **(F)**. **(G)** Nomogram predicting 1-, 3-, and 5- year OS of patients based on NRGscore and other clinical parameters, including gender, age, T stage, and N stage. The red dot presented the point of each parameter, and the length of the line segment reflected the contribution of factors to the outcome event. **(H)** Calibration plot of the nomogram for predicting the probability of 1-, 3-, and 5-year OS. The colored line was the fit line and represented the predicted value (the horizontal axis) corresponding to the actual value (the vertical axis). The gray diagonal was the ideal case. P < 0.01**; P < 0.001***. TCGA, the cancer genome atlas; ICGC, international cancer genome consortium; NRGscore, necroptosis-related gene score; NRGrisk, necroptosis-related gene risk; ROC, receiver operating characteristic curves; OS, overall survival.

Then, the prognostic value of the necroptosis scoring system in predicting patients’ survival outcomes was also estimated. We divided HCC patients into two training and testing cohorts randomly, including 300 cases in the training set, and 299 cases in the test set. K-M curves demonstrated that HCC patients in the high NRGscore group had lower OS rates than their counterparts in overall, training, and test sets (P < 0.001) (**Figures 5A, C**). Next, ROC curves were plotted to appraise the accuracy of the scoring system in predicting survival at 1, 3, and 5 years. (Overall set: AUC at 1-, 3-, and 5-year is 0.766, 0.718, and 0.685; Training set: 0.774, 0.756, and 0.824, respectively; Test set: 0.762, 0.691, and 0.530, separately; **Figures 5D, F**). Besides, we assessed the NRGscore of each HCC case amidst the overall, training, and test sets, which implied that HCC patients in the low NRGscore group had better survival status and less ratio of dead status than the high NRGscore group (**Figures S6B, D**). Therefore, a high NRGscore was considered a high NRGrisk while a low NRGscore was deemed a low NRGrisk. In addition, to further evaluate the clinical application of the NRGscore system, we downloaded an external HCC dataset (GSE54236) and calculated the NRGscore of each HCC patient. The survival curve demonstrated that the high NRGrisk had a poorer prognosis than the low risk (**Figure S7A**), and the ROC curve indicated the NRGscore system had a robust predictive ability (**Figure S7B**). We also found patients with high NRGrisk had advanced clinical stage and grade than low NRGrisk (**Table S5**). In general, the above results indicated that the necroptosis scoring system had a promising capacity to predict the survival of HCC patients.

To better build a convenient and applicable clinical prognosis evaluation method, we plotted a nomogram graph based on the NRGscore system and clinical features including gender, age, and pathologic stage (**Figure 5G**). The concordance index of the nomogram was 0.707 (95% CI 0.652–0.762). The calibration plot for the possibility of 1-, 3-, and 5-year survival exhibited good coincidence degrees of survival probability between the prediction and real observations (**Figure 5H**). These results demonstrated that the nomogram could be an effective approach to predicting the prognosis outcome of HCC patients for clinicians. All the above results disclosed that NRGscore could have a satisfying clinical prediction value in HCC.

### NRGscore Accurately Predicts Immunotherapeutic Benefits for HCC

**Figure 6:**
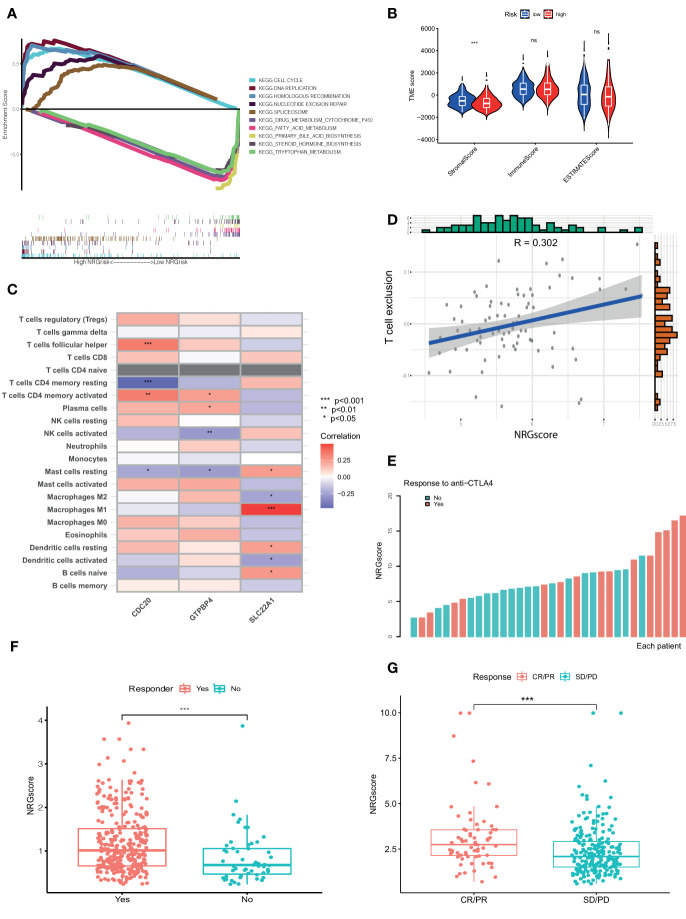
NRGscore could accurately predict response to ICBs. **(A)** Different biological features in high and low NRGrisk groups by GSEA in KEGG pathways. **(B)** Violin diagrams exhibited the correlation of immune, stromal, and ESTIMATE scores with NRGrisk groups. **(C)** Heatmap indicated the relevance of TICs and three hub genes in NRGscore. Different colors represented the different degrees of correlation. **(D)** T cell exclusion score was positively associated with NRGscore in the GSE54236 HCC cohort. **(E)** Waterfall diagram of NRGscore with responder or non-responder to CTLA-4 cohort. **(F)** Patients who respond to ICB possess more NRGscore than non-responders in the TCGA cohort by the TIDE algorithm. **(G)** NRGscore of patients varied with different responses to immunotherapy in the advanced urothelial cancer cohort. CR/PR is short for complete response or partial response; SD/PD represented the stable disease or progressive disease. P < 0.05 *; P < 0.01**; P < 0.001***. ICBs, immune checkpoint blockades; NRGscore, necroptosis-related gene score; TICs, tumor-infiltrating cells; NRGrisk, necroptosis-related gene risk; TIDE, tumor immune dysfunction, and exclusion.

Based on the above results, Ncluster A was characterized by more abundant immune infiltrating cells and enhanced immune function than Ncluster B. Ncluster A could represent the immune-inflamed type and B represent the immune-excluded phenotype, which gave us a hint that the Ncluster A subgroup of patients could respond better to immunotherapy than B cluster. As shown in the alluvial diagram, high NRGscore had an overwhelming ratio of Ncluster A while low NRGscore were nearly derived from Ncluster B. Therefore, we speculated that patients with high NRGscore could be more sensitive to immunotherapy. First, we performed GSEA to investigate the biological of high and low NRGrisk, and the results (**Figure 6A**) revealed that high NRGrisk was characterized by cell proliferation and DNA repair-related pathways (cell cycle, DNA replication, nucleotide exclusion repair, and spliceosome, et al.) while low NRGrisk was characterized by enhanced metabolism pathways (drug metabolism of P450, fatty acid metabolism, and bile acid metabolism, et al.). Alternatively, the above results were also verified by other famous pathways, including hallmark pathways (**Figure S8A**) and reactome pathways (**Figure S8B**). Surprisingly, almost all immune pathways were enriched in the high NRGrisk group (**Figure S8C**), which further demonstrated high NRGrisk resembled Ncluster A group and could be more sensitive to clinical immunotherapy. Additionally, we further calculated the TME parts, including stromal, immune, and ESTIMATE parts. As expected, the stromal component was significantly downregulated in the high NRGrisk score group (**Figure 6B**). Previous research underlined the hub role of stromal activation in resistance to checkpoint immunotherapy and high NRGrisk patients with low stromal could get more clinical benefits from immunotherapy. To comprehensively evaluate the correlation between the scoring system and immunotherapy, we first estimated the TICs abundance based on the NRGscore. Results indicated that core genes in the scoring system had a close relation to various types of TICs. Among them, CDC20 was mainly related to T immune cells; GTPBP4 had a tight correlation with NK immune cells; SLC22A1 was mostly correlated to macrophages and DC immune cells (**Figure 6C**). Various immune functions altered with the NRGscore system; the heatmap indicated that a higher NRGscore always followed more inhibition of immune response (APC co-inhibition, MHC-I class, T cell co-inhibition and Treg cell, IFN-response inhibition; **Figure S8D**). Besides, three TICs were significantly positively correlated with the NRGscore, including Mast cells activated (R=0.22), T cells follicular helper (R=0.26), and T cells CD4 memory activated (R=0.32), while five TICs were significantly negatively correlated, including B cells naïve (R=-0.24), NK cells activated (R=-0.24), macrophages M1 (R=-0.25), Mast cells resting (R=-0.3), and T cells CD4 memory resting (R=-0.36) (**Figure S9**). To our knowledge, PD-L expression was considered as an important predictor of response to ICBs, and we found that NRGscore was positively associated with several known immune checkpoints (PD-1, PD-L1, PD-L2, CTLA4, TIM-3, IDO1, LAG3, TIGIT; **Table S6**). Integrating the results above, we found the NRGscore system could be dramatically correlated to the immune landscape of HCC, and its role in estimating the immunotherapy deserved further exploration.

For the present limitation research, the clinical application of immunotherapy in HCC was still not widespread and there are few biomarkers to predict the response to ICBs (23). Therefore, we evaluated the response of checkpoint immunotherapy to patients with different NRGscore. In the cohort of GSE54236, we found NRGscore was positively linked to the level of T cell exclusion, which indicated a high NRGscore with strong immune inhibition and could respond to ICB therapy (**Figure 6D**). In another cohort of metastatic melanoma, patients with response to CTLA-4 therapy possessed a higher NRGscore (**Figure 6E**). In addition, we also found similar results in the TCGA HCC cohort (**Figure 6F**) and IMvigor210 cohort treated with atezolizumab (**Figure 6G**). IPS values were elevated in high NRGscore subgroups (**Table S7**), which indicated that the high NRGscore subgroup was relatively responsive to immunotherapy. In other cancer cohorts with ICB therapy, the NRGscore system could also accurately predict ICB response (**Figure S10**). Collectively, the above results elucidated that the NRGscore system could play a non-negligible role in predicting the immunotherapy response in HCC patients, or even in other tumors.

### Patients With High NRGrisk Were More Sensitive to Common Chemotherapy Regimens

**Figure 7:**
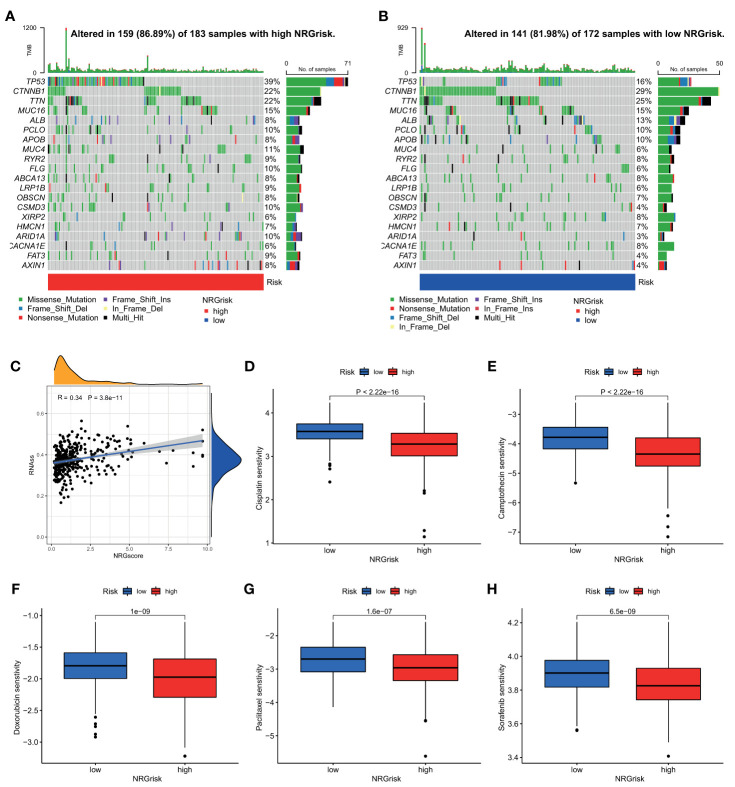
Characteristics of NRGscore with tumor somatic mutation, stem cell, and chemotherapy sensitivity. **(A, B)** The waterfall plot of tumor somatic mutation was drawn in those with high NRGrisk group **(A)** and low NRGrisk **(B)** respectively. Each column indicated individual HCC patients, and the upper bar diagram exhibited TMB. The right number represented the mutation frequency, and the bar diagram on the right exhibited the proportion of each variant type, including missense, nonsense, splice, frameshift, and multiple mutations. **(C)** Correlation between NRGscore and component of stem cell. **(D-H)** Box diagrams showed the chemotherapy response between high NRGrisk and low NRGrisk groups: **(D)** Cisplatin, **(E)** Camptothecin, **(F)** Doxorubicin, **(G)** Paclitaxel, **(H)** Sorafenib. NRGscore, necroptosis-related gene score; NRGrisk, necroptosis-related gene risk; HCC, hepatocellular carcinoma; TMB, tumor mutation burden; IC50, half-maximal inhibitory concentration.

As a widely accepted biomarker for prognosis and immunotherapy, tumor mutation burden (TMB) was also evaluated in our study. Although there were few differences in TMB between high and low NRGrisk subgroups (**Figure S11A**), and there is no linear correlation of TMB with NRGscore (**Figure S11B**), the gene mutation type altered in two subgroups in the waterfall curve (**Figures 7A, B**). Results indicated that the high NRGscore subgroup presented more extensive TMB than the low score subgroup. Among them, TP53 was the first mutated gene with a 39% mutation alteration rate in the high score subgroup, while CTNNB1 was the top rank mutated gene with a 29% mutation alteration rate in the low score subgroup (**Figures 7A,B**). Consistent with a recent study, CTNNB1, TP53 mutation-associated pathways, and metabolism profiles were identified in HBV-related HCC (55). Besides, we found that the ratio of tumor stem cells in HCC was significantly positively related to NRGscore (R=0.34) (**Figure 7C**). Considering the frequent use of chemotherapy in the treatment of HCC, we further explored the response of patients with 138 different types of drugs. Analyses of consequences revealed that several prevalent clinical chemotherapies of HCC exhibited low IC50 in the high NRGscore group, including Cisplatin, Camptothecin, Doxorubicin, Paclitaxel, and Sorafenib. The finding suggested that patients with high NRGscore were more sensitive to the treatment of chemotherapy drugs than those with low scores in HCC (**Figures 7D, H**).

## Discussion

The NRGscore, a tool designed to evaluate necroptosis patterns of each HCC patient, is a robust biomarker for predicting clinical outcomes and for guiding rational and effective immunotherapy. Our findings revealed a survival benefit trend was observed in Ncluster A compared to B, suggesting necroptosis played a non-negligible role in the progression and prognosis in HCC. GSVA analysis disclosed that Ncluster A had more TME-infiltrating cells and more complex immune-response than Ncluster B, substantiating Ncluster could be a surrogate and favorable biomarker for TME. However, individual necroptosis characteristics could not be elucidated dependent on Ncluster, and we built a novel NRGscore scheme, determined by three core genes (GTPBP4, CDC20, and SLC22A1). NRGscore could accurately and robustly predict 1-, 3-, and 5-year OS of HCC. More importantly, the high NRGscore group had more sensitivity to clinical chemotherapy and ICB-based immunotherapy than the low score group, which demonstrated the NRGscore could be meaningful guidance for HCC treatment.

Mounting evidence demonstrates that necroptosis plays a pivotal role in predicting clinical outcomes of cancer, regulating cancer progression and metastasis, remodeling TME, and thus affecting immunotherapy and chemotherapy (56). Although necroptosis had been shown to perform an antitumor function in cancers, much evidence also demonstrated that necroptosis may play a tumor-promoting role and trigger cancer metastasis (11, 57). The paradox phenomena could be explained by high inflammation TME caused by necroptosis and following elevated ROS levels could further promote cancer progression and metastasis (56). Our results uncovered that higher necroptosis patterns were, worse outcomes did HCC patients have due to most necroptotic regulators being risky genes in HCC. In addition, high NRGscore patients always had more mRNA levels of three necroptosis-driving genes (MLKL, RIPK1, and RIPK3), further validating that necroptosis could play a tumor-promoting role in HCC. In another research on liver cancer, necroptosis directly determines the cancer subtype by driving cell releasing damage-associated molecular patterns (DAMPs) which could reshape the TME (58), causing the switch from HCC to intrahepatic cholangiocarcinoma (ICC) (15). Here, we found that the Ncluster A subgroup had a cline to the high NRGscore group while Ncluster B resembled low NRGscore patients in the alluvial diagram. Ncluster A and high NRGscore were characterized by more TME-infiltrating cells and enhanced APC-co-inhibitor and checkpoint gene expression, corresponding to immune-inflamed phenotype. Ncluster B and low NRGscore were characterized by enhanced metabolism and decreased stem cells and proliferation, corresponding to immune-exclusion or metabolism phenotype. TIDE results suggested that the high NRGscore group with lower stromal activation could achieve clinical benefits in immunotherapy, consistent with research that emphasized the irreplaceable role of stromal activation in resistance to PD-1/PD-L1 inhibitors (45, 59, 60).

Furthermore, we elucidated the predictive value in another three independent cohorts (HCC cohort with TIDE results; metastatic melanoma cohort treated with Nivolumab; advanced urothelial cancer cohort with the intervention of atezolizumab (IMvigor210)). In line with the results of TCGA HCC, we observed a significant positive trend between NRGscore and T cell exclusion in the GSE54236 HCC cohort. In the metastatic melanoma cohort, responders to anti-CTLA4 possessed a higher NRGscore than non-responders. Similarly, patients’ sensitive to anti-PD-L1 had more NRGscore than non-responders. Furthermore, we input our NRGscore system into the TIDE website to predict other cancer cohorts undertaking ICBs and the results revealed that NRGscore could behave well in predicting response. We also found different somatic gene mutations in high and low NRGrisk groups and that TP53 mutation was most common in high risk and CTNNB1 for low risk. The results may help us to gain a comprehensive understanding of precision immunotherapy and promoting the necroptosis could enhance the response to ICBs. Intriguingly, we observed that NRGscore could perform as a candidate biomarker for classic chemotherapy and target therapy (Sorafenib, Doxorubicin, Cisplatin, Camptothecin, and Paclitaxel) for HCC.

Additionally, we have validated the pivotal role of three necroptosis-related genes in HCC. It has been widely accepted that the anaphase-promoting complex (APC) drives and governs cell cycle progression (anaphase initiation and late mitosis exit (61, 62)) by interacting with two essential activators - CDC20 and CDH1 (63). However, the two proteins performed different roles in tumor development. CDC20 was considered as an oncogene while CDH1 was deemed as a suppressor in multicancer (64–66). CDC20 has been reported to be overexpressed in HCC tissues and positively related to the tumor, TNM stage, and ki-67 expression (67). In tumorigenesis of HCC, APCCDC20 could stabilize the HIF-1a by degrading oxygen-dependent prolyl hydroxylase enzymes3 (PHD3) (68). Suppressing CDC20 (depletion of endogenous or pharmacological inhibitors) in diverse cancer cell lines led to a mitotic arrest and apoptosis, suggesting targeting CDC20 might be a novel anti-cancer therapy, especially in cancer with high expression (69). GTPBP was a GTP-binding protein that is involved in 60S ribosome biogenesis (70). The previous report found that GTPBP4 could reduce TP53 accumulation and increased expression of GTPBP4 correlated with reduced survival (71). In colorectal cancer, GTPBP4 was demonstrated as an oncogene that could disrupt the actin cytoskeleton and promote metastasis of cancer (72). In addition, depletion of GTPBP4 could inhibit cell proliferation, and high expression correlated with a worse prognosis than the low expression of GTPBP4 in breast cancer (73). In a study of HCC, as an RNA binding protein, GTPBP4 could stratify patients into low- and high-risk, which could predict survival outcomes (74). The upregulated expression of GTPBP4 promotes the proliferation of liver cancer cells and promotes the growth of tumors in mice, while the downregulated expression of GTPBP4 inhibits the proliferation of liver cancer cells and inhibits the growth of tumors in mice (75). SLC22A1 (OCT1) is one member of organic cation transporters, which could uptake intracellular inactivation, a broad spectrum of endogenous and exogenous substrates as well as anticancer drugs (76–78). The OCT1 activity was reported to correlate with the sensitivity of tyrosine kinase inhibitors (TKI) in patients with chronic myeloid leukemia (CML) (79). The downregulation of OCT1 is associated with tumor progression and a worse patient survival (80).

The results of our study should be further validated in a prospective cohort of HCC patients undergoing immunotherapy or chemotherapy. Also, these three hub genes in NRGscore and their relationship with necroptosis should be further explored in basic experiments. Since not all high NRGscore patients would benefit from immunotherapy, more clinical parameters and appropriate evaluations of immune infiltration and TMB should be taken into consideration.

In the current study, we found that necroptosis patterns could become a novel index to predict HCC patients’ outcomes and high necroptosis levels might suggest a poor survival prognosis. Ncluster was built based on these necroptosis regulators, and two Nclusters had immense changes in prognosis; cell phagocytosis-, endocytosis-, and peroxisome-related pathways; TME cell infiltration characterization; and immune function. In addition, we designed a novel individual necroptosis pattern evaluation system: NRGscore scheme. NRGscore could accurately and robustly predict 1-, 3-, and 5-year OS, which was validated in another HCC cohort. The novel nomogram was comprised of NRGscore and other clinicopathological features to visualize the risk of HCC patients. In ICB-based immunotherapy of HCC, NRGscore had meaningful guidance for patients and the high NRGscore group could be more sensitive to ICB treatment, which was further validated in the metastatic melanoma cohort and advanced urothelial cancer cohort. The high NRGscore group had an increased ratio of cancer stem cells than its counterpart, which further demonstrates high NRGscore could be more sensitive to chemotherapy and targeted molecular therapy.

## Conclusions

In the study, we found high expression of necroptosis could affect the TME of HCC and be closely related to the immune-inflamed phenotype. In predicting response to ICB, we concluded that high NRGrisk patients could be more sensitive than low NRGrisk. We systematically characterized the landscapes of necroptosis in HCC patients and suggested that NRGscore could be a prognostic marker and help to interpret the responses of HCC to chemotherapies and immunotherapies, providing new strategies for the treatment of cancers.

## Data Availability Statement

The original contributions presented in the study are included in the article/**Supplementary Material**. Further inquiries can be directed to the corresponding authors.

## Author Contributions

LL and YX designed the study. JZ and TH performed most of the results and completed the manuscript together. SZ, YZ, and SM helped with all experiments and data analysis. FX, TB, and YT helped write the manuscript. All authors read and approved the final manuscript.

## Funding

This work was supported by the National Natural Science Foundation of China (8217113876, 62171365).

## Conflict of Interest

The authors declare that the research was conducted in the absence of any commercial or financial relationships that could be construed as a potential conflict of interest.

## Publisher’s Note

All claims expressed in this article are solely those of the authors and do not necessarily represent those of their affiliated organizations, or those of the publisher, the editors and the reviewers. Any product that may be evaluated in this article, or claim that may be made by its manufacturer, is not guaranteed or endorsed by the publisher.
