# Supplementary material for: Computational Characterizing Necroptosis Reveals Implications for Immune Infiltration and Immunotherapy of Hepatocellular Carcinoma
Source: Front Oncol. 2022 Jul 7;12:933210. doi: 10.3389/fonc.2022.933210 (PMC9301124; doi:10.3389/fonc.2022.933210)
Supplement: Supplementary file 1 [file DataSheet_1.pdf]

Supplementary Figures S1-S11

Computational characterizing necroptosis reveals implications for immune infiltration and immunotherapy of hepatocellular carcinoma

Jun Zhu[1, 2#], Tenghui Han[3#], Shoujie Zhao[4], Yejing Zhu[4], Shouzheng Ma[5], Fenghua Xu[1], Tingting Bai[1], Yuxin Tang[1], Yungang Xu[6,7*], Lei Liu[1*]

[1] Department of Gastroenterology, Daping Hospital, Army Medical University, Chongqing 40042, China
[2] Department of General Surgery, The Southern Theater Air Force Hospital, Guangzhou, 510000, China.
[3] Department of Neurology, Xijing Hospital, Fourth Military Medical University, Xi'an, 710000, China.
[4] Department of General Surgery, Tangdu Hospital, Fourth Military Medical University, Xi'an710038, China
[5] Department of Surgery, Tangdu Hospital, Fourth Military Medical University, Xi'an, 710038, China
[6] Department of Cell Biology and Genetics, School of Basic Medical Sciences, Xi'an Jiaotong University Health Science Center, Xi'an 710061, China
[7] Centre for Computational Systems Medicine, School of Biomedical Informatics, The University of Texas Health Science Centre at Houston, TX 77030, USA

* To whom correspondence should be addressed. Email: liulei84207@tmmu.edu.cn, yungang.xu@uth.tmc.edu.

Figure S1. The workflow of this study.

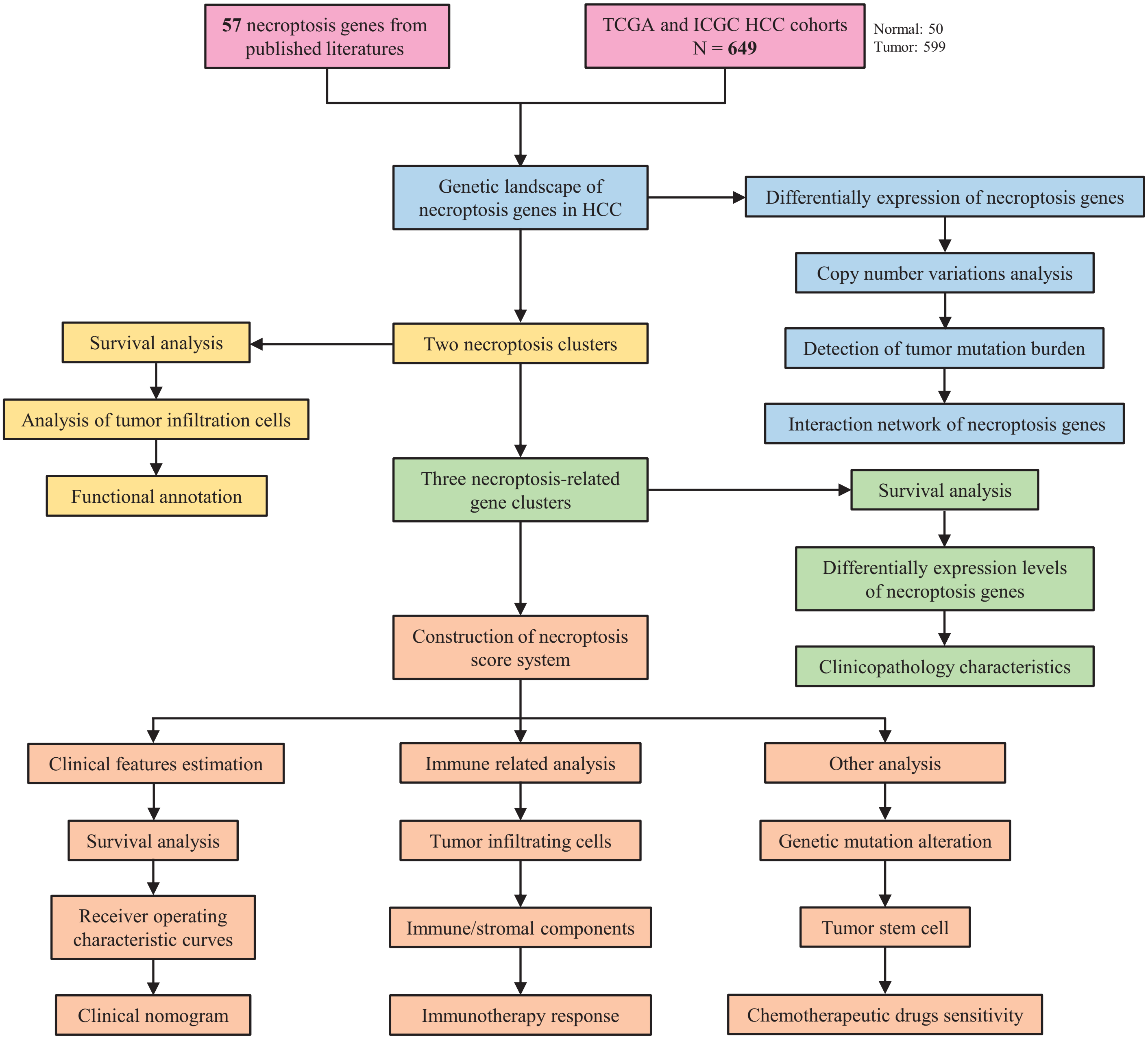
57 necroptosis genes from published literatures
TCGA and ICGC HCC cohorts N = 649
Normal: 50
Tumor: 599
Genetic landscape of necroptosis genes in HCC
Differentially expression of necroptosis genes
Copy number variations analysis
Detection of tumor mutation burden
Interaction network of necroptosis genes
Two necroptosis clusters
Survival analysis
Analysis of tumor infiltration cells
Functional annotation
Three necroptosis-related gene clusters
Survival analysis
Differentially expression levels of necroptosis genes
Clinicopathology characteristics
Construction of necroptosis score system
Clinical features estimation
Survival analysis
Receiver operating characteristic curves
Clinical nomogram
Immune related analysis
Tumor infiltrating cells
Immune/stromal components
Immunotherapy response
Other analysis
Genetic mutation alteration
Tumor stem cell
Chemotherapeutic drugs sensitivity

Figure S2. Correlation between two Nclusters for clinical features.

Clinical information included age, gender, grade, and stage. Most necroptosis genes were upregulated in Ncluster A. Ncluster: necroptosis cluster.

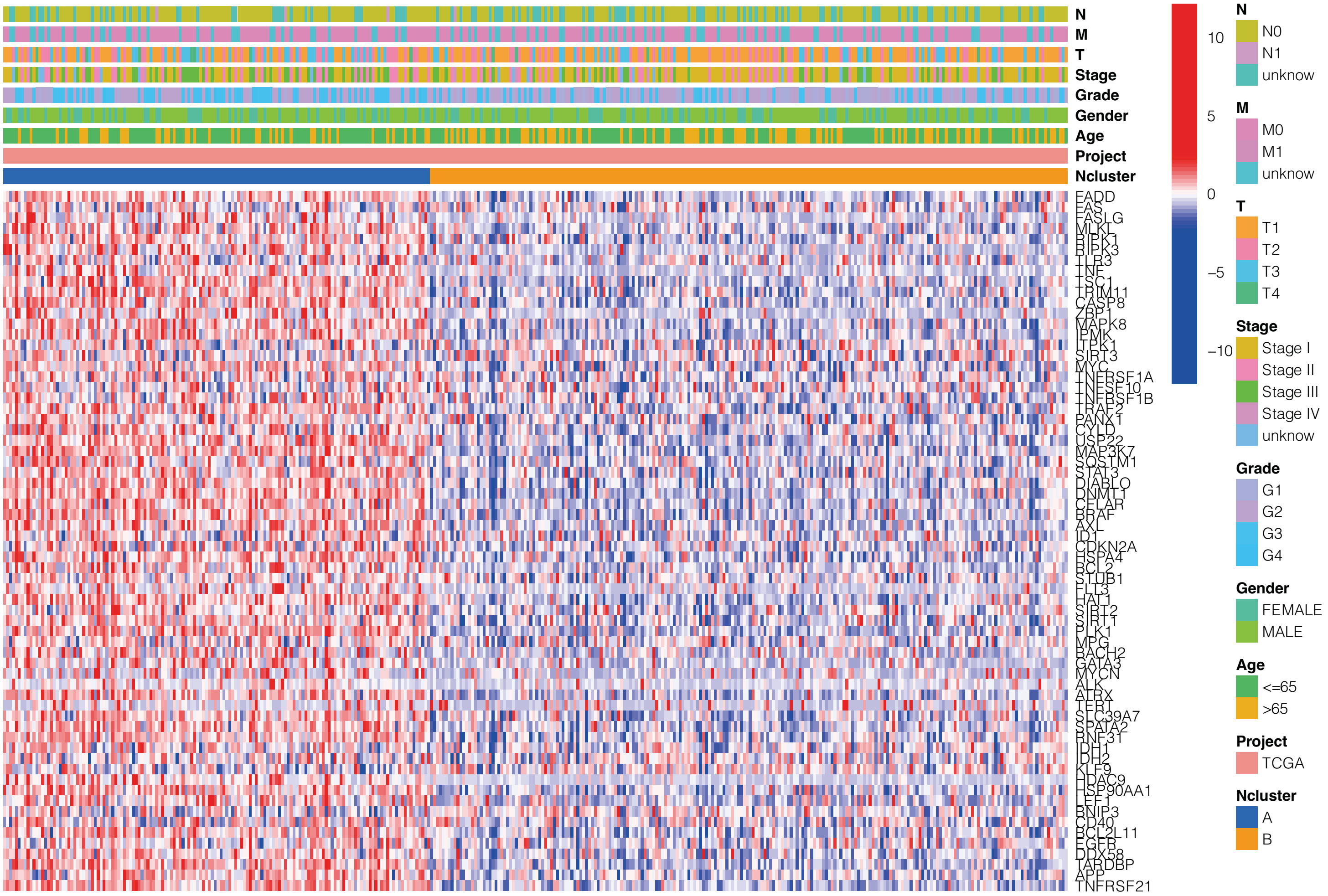
N
M
T
Stage
Grade
Gender
Age
Project
Ncluster
FADD
FAS
FASLG
MLKL
RIPK1
RIPK3
TLR3
TNF
TSC1
TRIM11
CASP8
ZBP1
MAPK8
IPMK
ITPK1
SIRT3
MYC
TNFRSF1A
TNFSF10
TNFRSF1B
TRAF2
PANX1
CYLD
USP22
MAP3K7
SQSTM1
STAT3
DIABLO
DNMT1
CFLAR
BRAF
AXL
ID1
CDKN2A
HSPA4
BCL2
STUB1
FLT3
HAT1
SIRT2
SIRT1
PLK1
MPG
BACH2
GATA3
MYCN
ALK
ATRX
TERT
SLC39A7
SPATA2
RNF31
IDH1
IDH2
KLF9
HDAC9
HSP90AA1
LEF1
BNIP3
CD40
BCL2L11
EGFR
DDX58
TARDBP
APP
TNFRSF21
10
5
0
−5
−10
N
N0
N1
unknow
M
M0
M1
unknow
T
T1
T2
T3
T4
Stage
Stage I
Stage II
Stage III
Stage IV
unknow
Grade
G1
G2
G3
G4
Gender
FEMALE
MALE
Age
<=65
>65
Project
TCGA
Ncluster
A
B

Figure S3. Functional enrichment analysis in two Nclusters.

(A) Immune-related pathways were differentially enriched in Ncluster A and B. **(B)** GO enrichment analysis of DEGs between two Nclusters: BP is short for biological processes; CC is the cellular component; MF is the molecular function. Length of column represented the enrichment DEG count; the size of points represented the enrichment DEG ratio; different colors represented the different q-values. **(C)** KEGG enrichment analysis of DEGs between two clusters. Nclusters, necroptosis clusters; GO, gene ontology; DEG, differential expression gene; KEGG, Kyoto encyclopedia of genes and genomes.

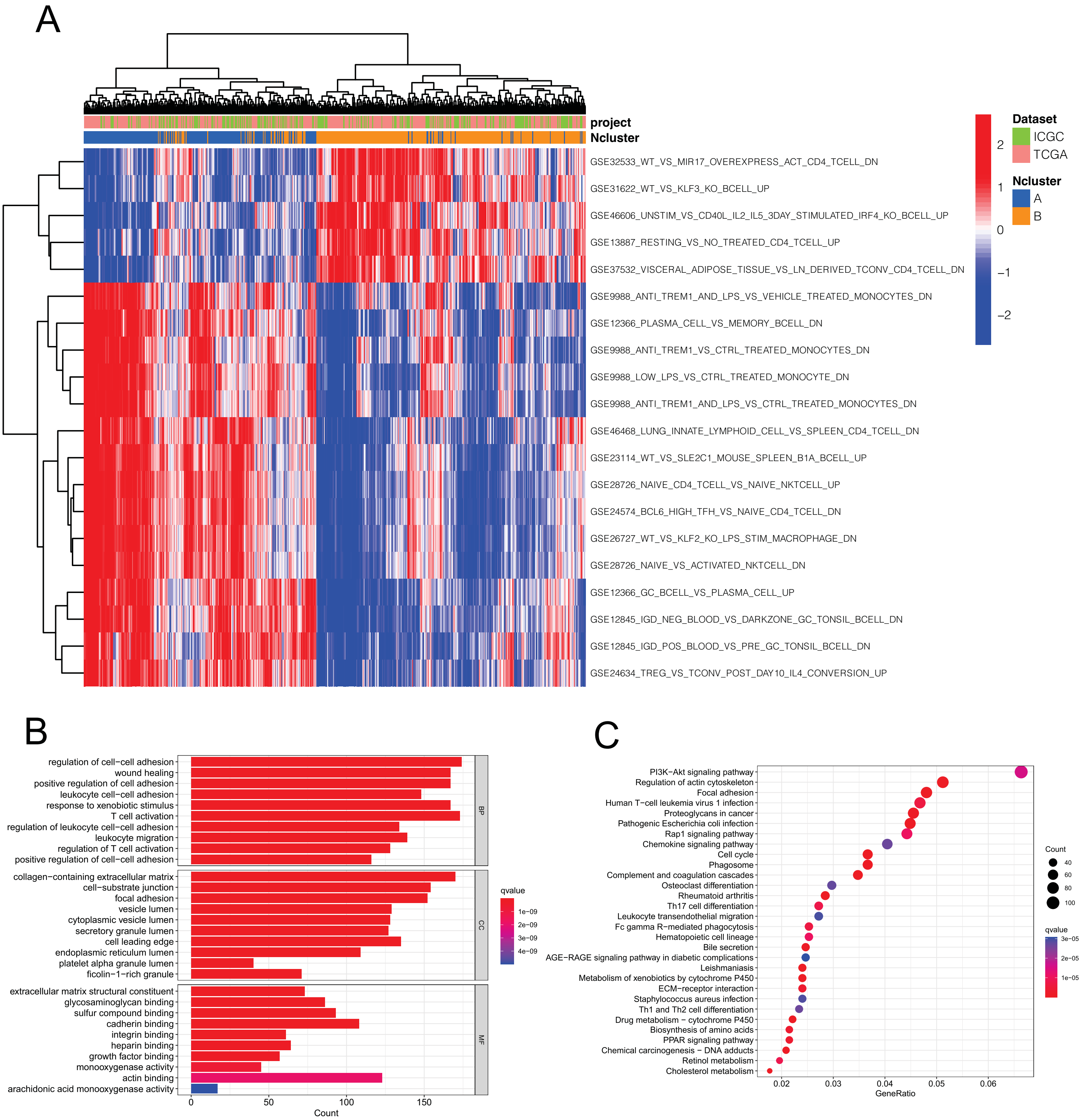
A
project
Ncluster
GSE32533_WT_VS_MIR17_OVEREXPRESS_ACT_CD4_TCELL_DN
GSE31622_WT_VS_KLF3_KO_BCELL_UP
GSE46606_UNSTIM_VS_CD40L_IL2_IL5_3DAY_STIMULATED_IRF4_KO_BCELL_UP
GSE13887_RESTING_VS_NO_TREATED_CD4_TCELL_UP
GSE37532_VISCERAL_ADIPOSE_TISSUE_VS_LN_DERIVED_TCONV_CD4_TCELL_DN
GSE9988_ANTI_TREM1_AND_LPS_VS_VEHICLE_TREATED_MONOCYTES_DN
GSE12366_PLASMA_CELL_VS_MEMORY_BCELL_DN
GSE9988_ANTI_TREM1_VS_CTRL_TREATED_MONOCYTES_DN
GSE9988_LOW_LPS_VS_CTRL_TREATED_MONOCYTE_DN
GSE9988_ANTI_TREM1_AND_LPS_VS_CTRL_TREATED_MONOCYTES_DN
GSE46468_LUNG_INNATE_LYMPHOID_CELL_VS_SPLEEN_CD4_TCELL_DN
GSE23114_WT_VS_SLE2C1_MOUSE_SPLEEN_B1A_BCELL_UP
GSE28726_NAIVE_CD4_TCELL_VS_NAIVE_NKTCELL_UP
GSE24574_BCL6_HIGH_TFH_VS_NAIVE_CD4_TCELL_DN
GSE26727_WT_VS_KLF2_KO_LPS_STIM_MACROPHAGE_DN
GSE28726_NAIVE_VS_ACTIVATED_NKTCELL_DN
GSE12366_GC_BCELL_VS_PLASMA_CELL_UP
GSE12845_IGD_NEG_BLOOD_VS_DARKZONE_GC_TONSIL_BCELL_DN
GSE12845_IGD_POS_BLOOD_VS_PRE_GC_TONSIL_BCELL_DN
GSE24634_TREG_VS_TCONV_POST_DAY10_IL4_CONVERSION_UP
2
1
0
−1
−2
Dataset
ICGC
TCGA
Ncluster
A
B
B
regulation of cell−cell adhesion
wound healing
positive regulation of cell adhesion
leukocyte cell−cell adhesion
response to xenobiotic stimulus
T cell activation
regulation of leukocyte cell−cell adhesion
leukocyte migration
regulation of T cell activation
positive regulation of cell−cell adhesion
collagen−containing extracellular matrix
cell−substrate junction
focal adhesion
vesicle lumen
cytoplasmic vesicle lumen
secretory granule lumen
cell leading edge
endoplasmic reticulum lumen
platelet alpha granule lumen
ficolin−1−rich granule
extracellular matrix structural constituent
glycosaminoglycan binding
sulfur compound binding
cadherin binding
integrin binding
heparin binding
growth factor binding
monooxygenase activity
actin binding
arachidonic acid monooxygenase activity
BP
CC
MF
qvalue
1e−09
2e−09
3e−09
4e−09
0
50
100
150
Count
C
PI3K−Akt signaling pathway
Regulation of actin cytoskeleton
Focal adhesion
Human T−cell leukemia virus 1 infection
Proteoglycans in cancer
Pathogenic Escherichia coli infection
Rap1 signaling pathway
Chemokine signaling pathway
Cell cycle
Phagosome
Complement and coagulation cascades
Osteoclast differentiation
Rheumatoid arthritis
Th17 cell differentiation
Leukocyte transendothelial migration
Fc gamma R−mediated phagocytosis
Hematopoietic cell lineage
Bile secretion
AGE−RAGE signaling pathway in diabetic complications
Leishmaniasis
Metabolism of xenobiotics by cytochrome P450
ECM−receptor interaction
Staphylococcus aureus infection
Th1 and Th2 cell differentiation
Drug metabolism − cytochrome P450
Biosynthesis of amino acids
PPAR signaling pathway
Chemical carcinogenesis − DNA adducts
Retinol metabolism
Cholesterol metabolism
0.02
0.03
0.04
0.05
0.06
GeneRatio
Count
40
60
80
100
qvalue
3e−05
2e−05
1e−05

Figure S4. Clinical features and differentially expression analysis of NRGclusters.

(A) The heatmap exhibited the correlation of three NRGclusters and clinical characteristics. Clinical information included age, gender, grade, and stage. **(B)** Distinct expression levels of 57 necroptosis regulators in three genomic clusters. $P < 0.05$ *; $P < 0.01$**; $P < 0.001$***. NRGclusters, necroptosis-related genes clusters.

A

B

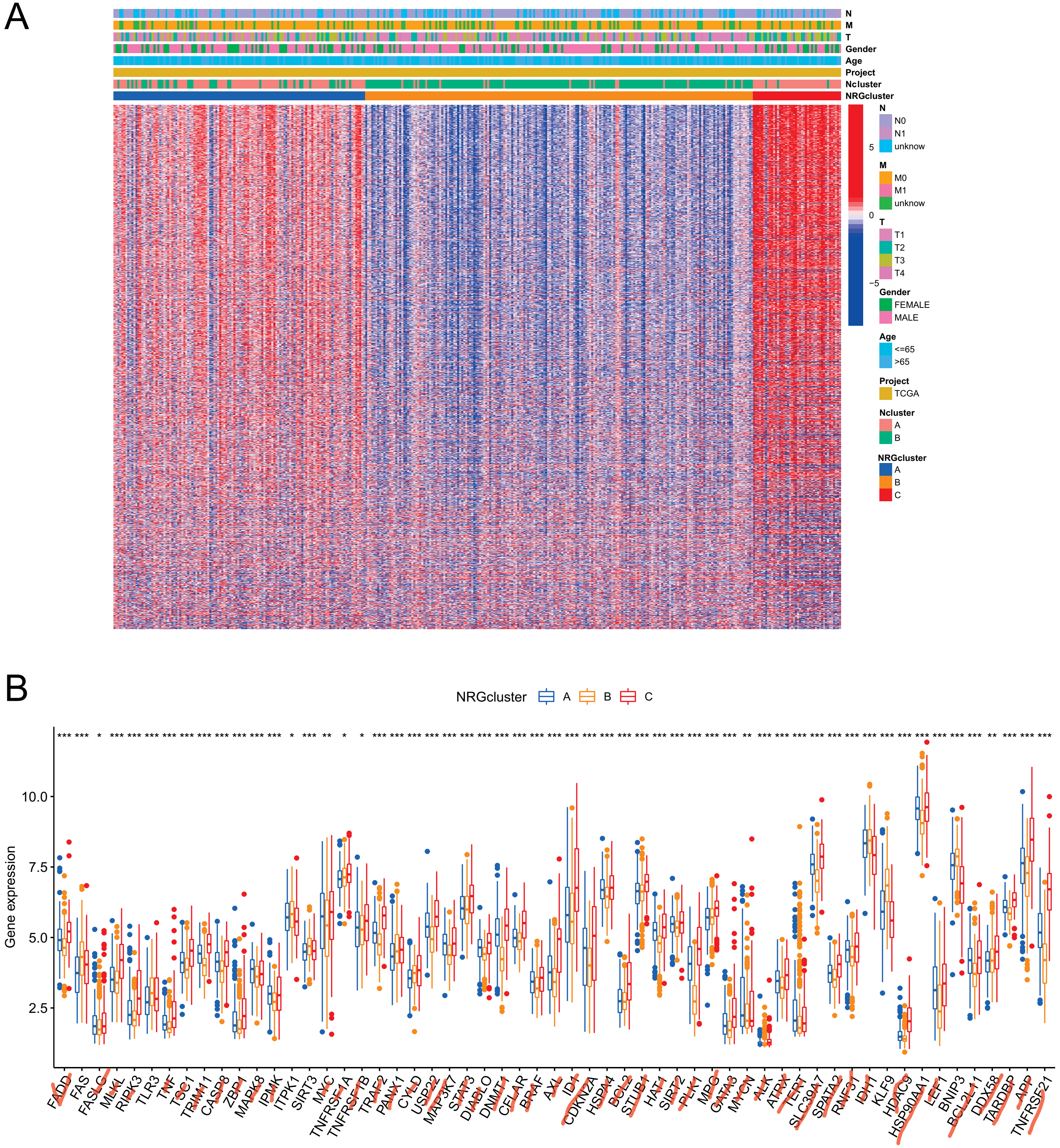
N
M
T
Gender
Age
Project
Ncluster
NRGcluster
5
0
−5
N0
N1
unknow
M0
M1
unknow
T1
T2
T3
T4
FEMALE
MALE
<=65
>65
TCGA
A
B
C
NRGcluster A B C
Gene expression
10.0
7.5
5.0
2.5
FADD FAS FASLG MLKL RIPK3 TLR3 TNF TSC1 TRIM11 CASP8 ZBP1 MAPK8 IPMK ITPK1 SIRT3 MYC TNFRSF1A TNFRSF1B TRAF2 PANX1 CYLD USP22 MAP3K7 STAT3 DIABLO DNMT1 CFLAR BRAF AXL ID1 CDKN2A HSPA4 BCL2 STUB1 HAT1 SIRT2 PLK1 MPO GATA3 MYCN ALK ATRX TERT SLC39A7 SPATA2 RNF31 IDH1 KLF9 HDAC9 HSP90AA1 LEF1 BNIP3 BCL2L11 DDX58 TARDBP APP TNFRSF21

Figure S5. Relationship between NRGscore and three main necroptosis-driving regulators.

(A) MLKL; **(B)** RIPK1; **(C)** RIPK3. NRGscore, necroptosis-related gene score.

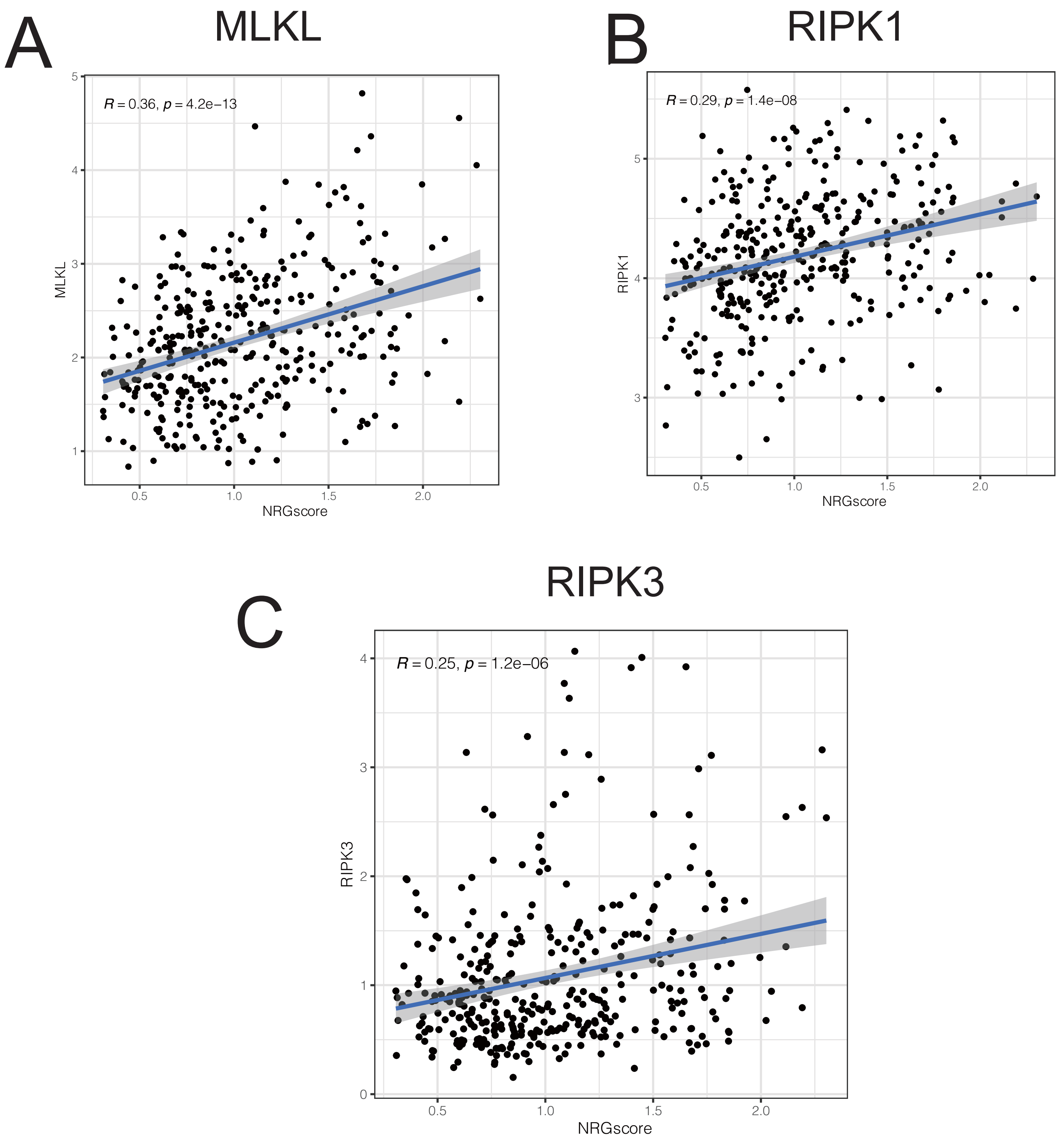
A
MLKL
R = 0.36, p = 4.2e−13
MLKL
NRGscore
B
RIPK1
R = 0.29, p = 1.4e−08
RIPK1
NRGscore
C
RIPK3
R = 0.25, p = 1.2e−06
RIPK3
NRGscore

Figure S6. Modelling characteristics of NRGscore.

(A) Necroptosis-related genes expression between two different NRGscore groups via differential expression analysis. **(B-D)** Distributions of NRGscore and survival status of HCC patients in the overall set (B), train set (C), and test set (D). HCC, hepatocellular carcinoma; NRGscore, necroptosis-related gene score.

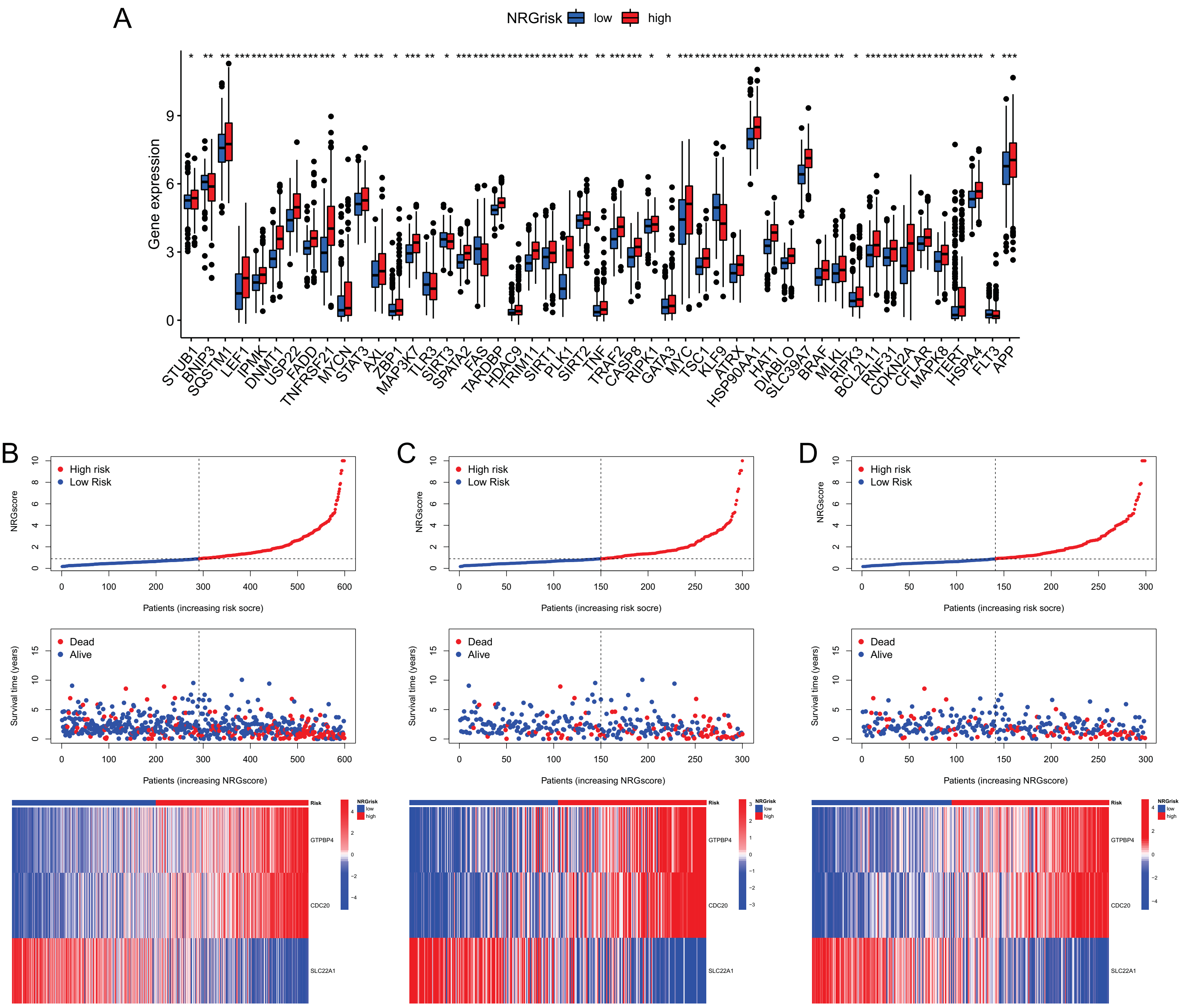
A
NRGrisk
low
high
Gene expression
STUB1
BNIP3
SQSTM1
LEF1
IPMK
DNMT1
USP22
FADD
TNFRSF21
MYCN
STAT3
AXL
ZBP1
MAP3K7
TLR3
SIRT3
SPATA2
FAS
TARDBP
HDAC9
TRIM11
SIRT1
PLK1
SIRT2
TNF
TRAF2
CASP8
RIPK1
GATA3
MYC
TSC1
KLF9
ATRX
HSP90AA1
HAT1
DIABLO
SLC39A7
BRAF
MLKL
RIPK3
BCL2L11
RNF31
CDKN2A
CFLAR
MAPK8
TERT
HSPA4
FLT3
APP
B
C
D
High risk
Low Risk
NRGscore
Patients (increasing risk socre)
Dead
Alive
Survival time (years)
Patients (increasing NRGscore)
Risk
NRGrisk
low
high
GTPBP4
CDC20
SLC22A1

Figure S7. Validation of NRGscore system in another HCC cohort (GSE54236)

(A) Survival curve between high and low NRGrisk groups. **(B)** ROC curve to assess the predictive performance of NRGscore.

A

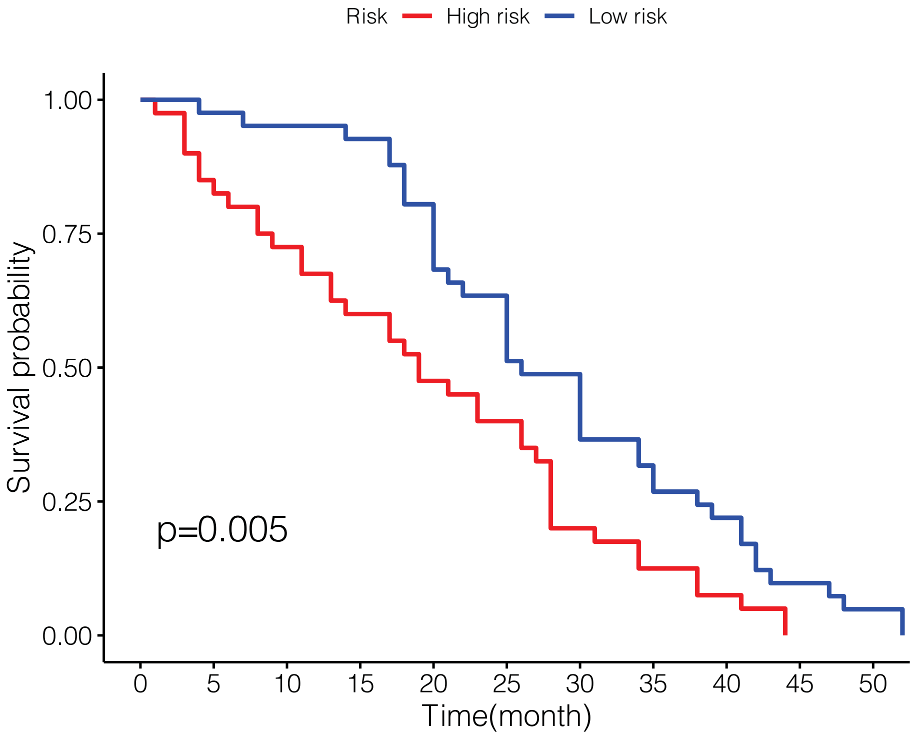
Risk
High risk
Low risk
Survival probability
1.00
0.75
0.50
0.25
0.00
p=0.005
0
5
10
15
20
25
30
35
40
45
50
Time(month)

B

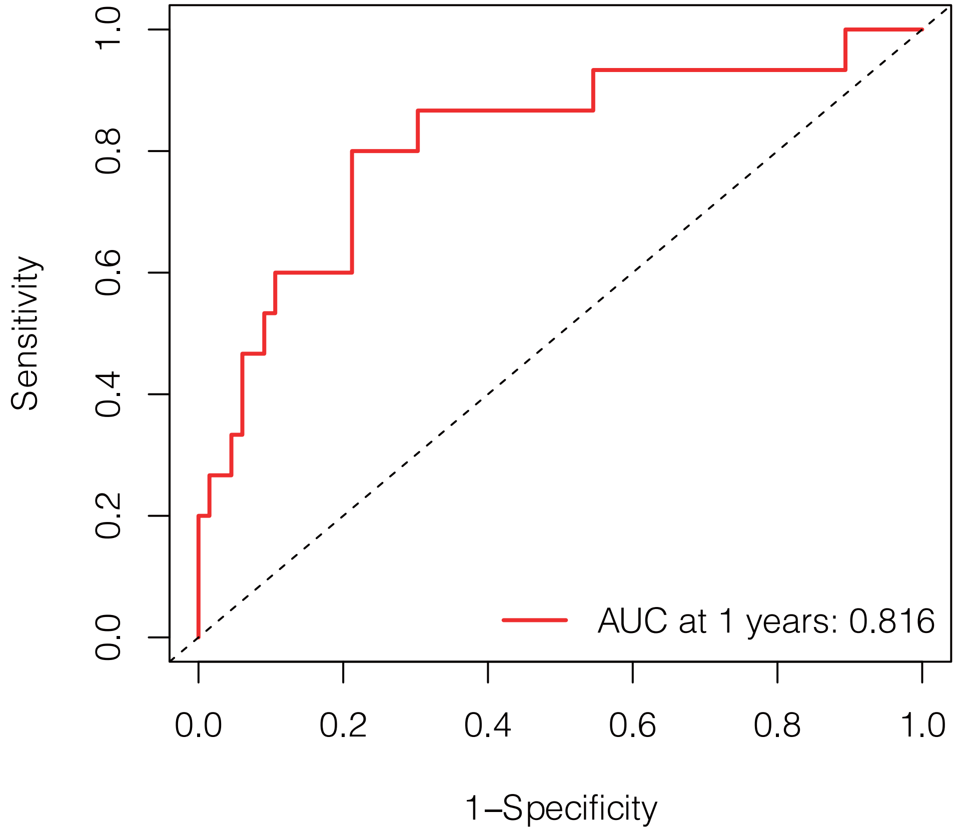
Sensitivity
0.0
0.2
0.4
0.6
0.8
1.0
AUC at 1 years: 0.816
1–Specificity

Figure S8. Biological and immune features between high and low NRGrisk groups.

(A) Hallmark pathways, **(B)** Reactome pathways, and **(C)** immune pathways were differentially enriched in two NRGrisk groups by the GSEA algorithm. **(D)** NRGscore with immune function and common infiltrating cells by ssGSEA algorithm.

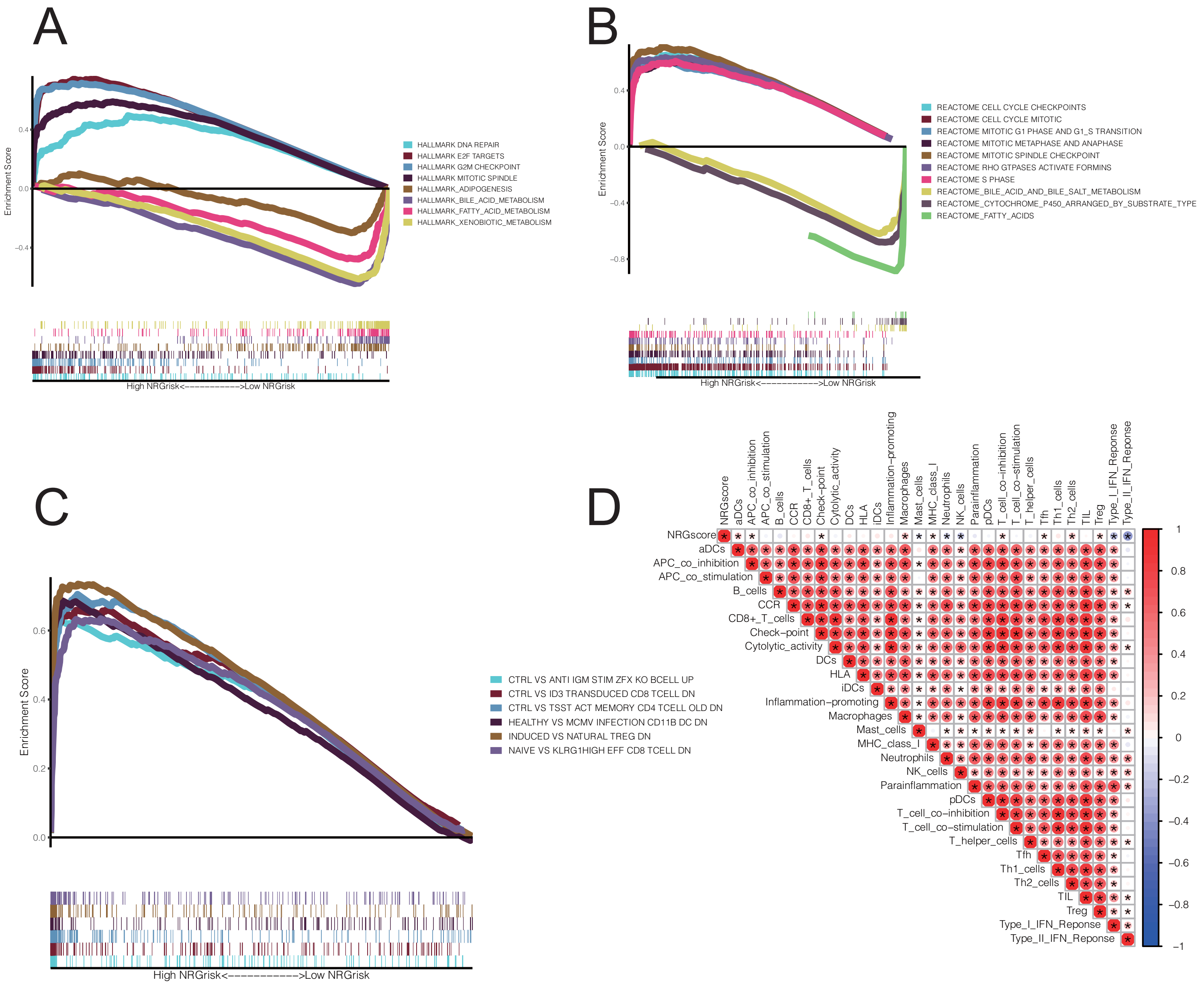
A
Enrichment Score
0.4
0.0
−0.4
HALLMARK DNA REPAIR
HALLMARK E2F TARGETS
HALLMARK G2M CHECKPOINT
HALLMARK MITOTIC SPINDLE
HALLMARK_ADIPOGENESIS
HALLMARK_BILE_ACID_METABOLISM
HALLMARK_FATTY_ACID_METABOLISM
HALLMARK_XENOBIOTIC_METABOLISM
High NRGrisk<----------->Low NRGrisk
B
Enrichment Score
0.4
0.0
−0.4
−0.8
REACTOME CELL CYCLE CHECKPOINTS
REACTOME CELL CYCLE MITOTIC
REACTOME MITOTIC G1 PHASE AND G1_S TRANSITION
REACTOME MITOTIC METAPHASE AND ANAPHASE
REACTOME MITOTIC SPINDLE CHECKPOINT
REACTOME RHO GTPASES ACTIVATE FORMINS
REACTOME S PHASE
REACTOME_BILE_ACID_AND_BILE_SALT_METABOLISM
REACTOME_CYTOCHROME_P450_ARRANGED_BY_SUBSTRATE_TYPE
REACTOME_FATTY_ACIDS
High NRGrisk<----------->Low NRGrisk
C
Enrichment Score
0.6
0.4
0.2
0.0
CTRL VS ANTI IGM STIM ZFX KO BCELL UP
CTRL VS ID3 TRANSDUCED CD8 TCELL DN
CTRL VS TSST ACT MEMORY CD4 TCELL OLD DN
HEALTHY VS MCMV INFECTION CD11B DC DN
INDUCED VS NATURAL TREG DN
NAIVE VS KLRG1HIGH EFF CD8 TCELL DN
High NRGrisk<----------->Low NRGrisk
D
NRGscore
aDCs
APC_co_inhibition
APC_co_stimulation
B_cells
CCR
CD8+_T_cells
Check−point
Cytolytic_activity
DCs
HLA
iDCs
Inflammation−promoting
Macrophages
Mast_cells
MHC_class_I
Neutrophils
NK_cells
Parainflammation
pDCs
T_cell_co−inhibition
T_cell_co−stimulation
T_helper_cells
Tfh
Th1_cells
Th2_cells
TIL
Treg
Type_I_IFN_Reponse
Type_II_IFN_Reponse
1
0.8
0.6
0.4
0.2
0
−0.2
−0.4
−0.6
−0.8
−1

Figure S9. Association of TICs and NRGscore.

Significant association of TICs and NRGscore: **(A)** Mast cells activated; **(B)** T cells follicular helper; **(C)** T cells CD4 memory activated, **(D)** B cells naïve, **(E)** NK cells activated, **(F)** Macrophages M1, **(G)** Mast cells resting, and **(H)** T cells CD4 memory resting. TICs, tumor-infiltrating cells; NRGscore, necroptosis-related gene score; NK cells, natural killer cells.

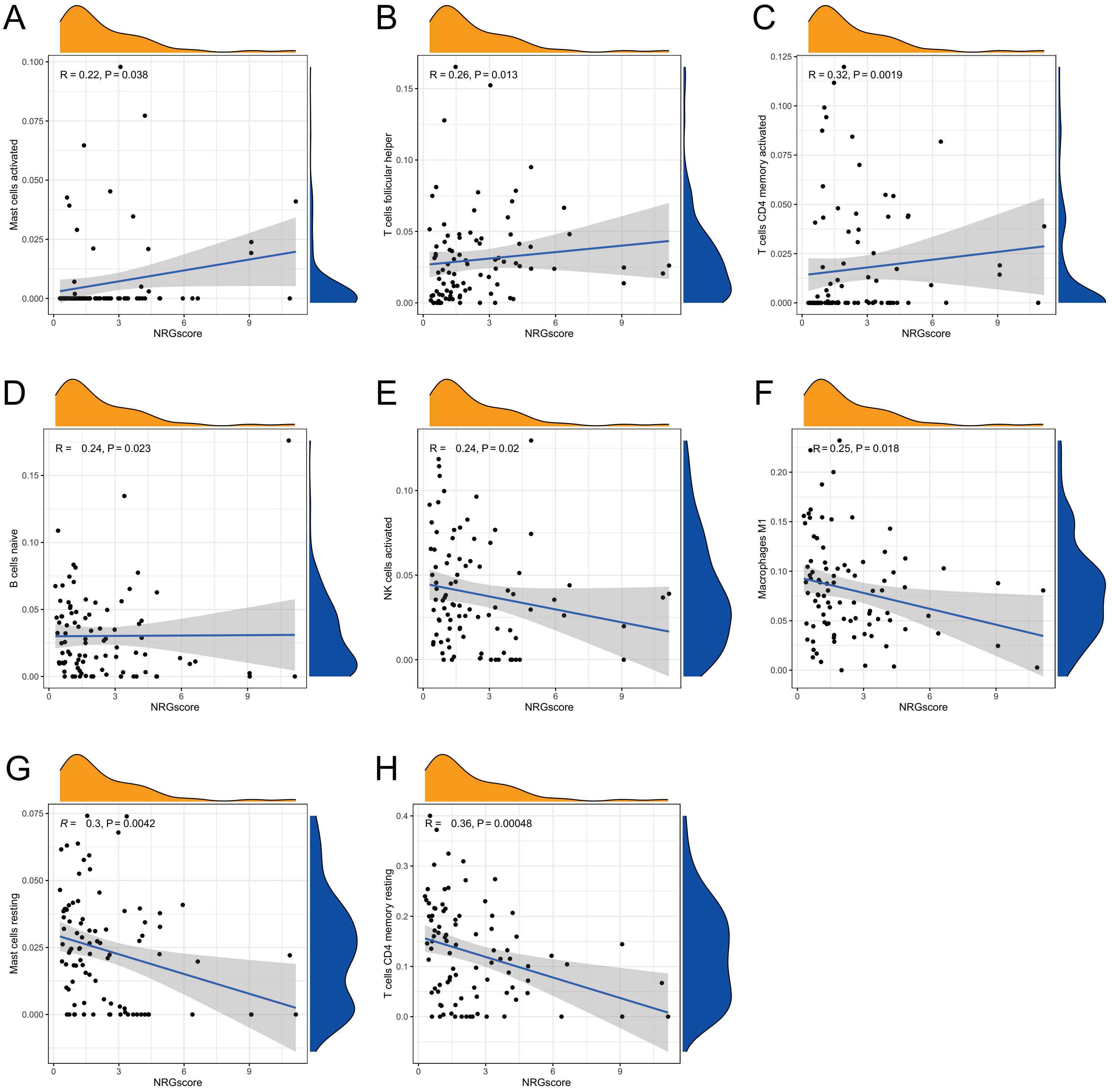

A
R = 0.22, P = 0.038
Mast cells activated
NRGscore
B
R = 0.26, P = 0.013
T cells follicular helper
NRGscore
C
R = 0.32, P = 0.0019
T cells CD4 memory activated
NRGscore
D
R = 0.24, P = 0.023
B cells naive
NRGscore
E
R = 0.24, P = 0.02
NK cells activated
NRGscore
F
R = 0.25, P = 0.018
Macrophages M1
NRGscore
G
R = 0.3, P = 0.0042
Mast cells resting
NRGscore
H
R = 0.36, P = 0.00048
T cells CD4 memory resting
NRGscore

Figure S10. Predictive performance of NRGscore in other cancer cohorts determined by the TIDE website.

TIDE, Tumor immune dysfunction, and exclusion.

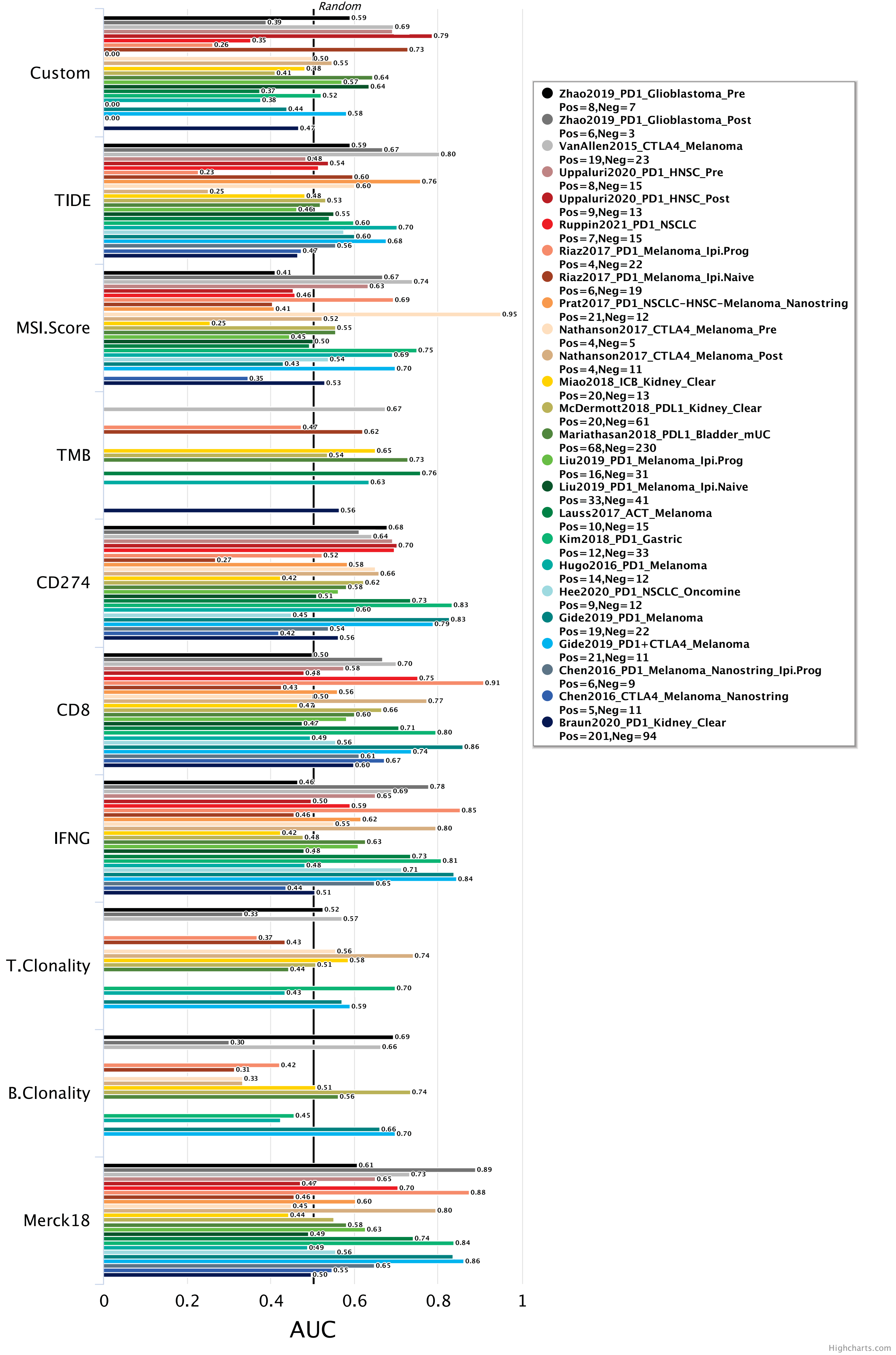

Random
Custom
TIDE
MSI.Score
TMB
CD274
CD8
IFNG
T.Clonality
B.Clonality
Merck18
0
0.2
0.4
0.6
0.8
1
AUC
Zhao2019_PD1_Glioblastoma_Pre Pos=8,Neg=7
Zhao2019_PD1_Glioblastoma_Post Pos=6,Neg=3
VanAllen2015_CTLA4_Melanoma Pos=19,Neg=23
Uppaluri2020_PD1_HNSC_Pre Pos=8,Neg=15
Uppaluri2020_PD1_HNSC_Post Pos=9,Neg=13
Ruppin2021_PD1_NSCLC Pos=7,Neg=15
Riaz2017_PD1_Melanoma_Ipi.Prog Pos=4,Neg=22
Riaz2017_PD1_Melanoma_Ipi.Naive Pos=6,Neg=19
Prat2017_PD1_NSCLC-HNSC-Melanoma_Nanostring Pos=21,Neg=12
Nathanson2017_CTLA4_Melanoma_Pre Pos=4,Neg=5
Nathanson2017_CTLA4_Melanoma_Post Pos=4,Neg=11
Miao2018_ICB_Kidney_Clear Pos=20,Neg=13
McDermott2018_PDL1_Kidney_Clear Pos=20,Neg=61
Mariathasan2018_PDL1_Bladder_mUC Pos=68,Neg=230
Liu2019_PD1_Melanoma_Ipi.Prog Pos=16,Neg=31
Liu2019_PD1_Melanoma_Ipi.Naive Pos=33,Neg=41
Lauss2017_ACT_Melanoma Pos=10,Neg=15
Kim2018_PD1_Gastric Pos=12,Neg=33
Hugo2016_PD1_Melanoma Pos=14,Neg=12
Hee2020_PD1_NSCLC_Oncomine Pos=9,Neg=12
Gide2019_PD1_Melanoma Pos=19,Neg=22
Gide2019_PD1+CTLA4_Melanoma Pos=21,Neg=11
Chen2016_PD1_Melanoma_Nanostring_Ipi.Prog Pos=6,Neg=9
Chen2016_CTLA4_Melanoma_Nanostring Pos=5,Neg=11
Braun2020_PD1_Kidney_Clear Pos=201,Neg=94
Highcharts.com

Figure S11. TMB alternation in two NRGrisk groups and three NRGclusters.

TMB, tumor mutation burden.

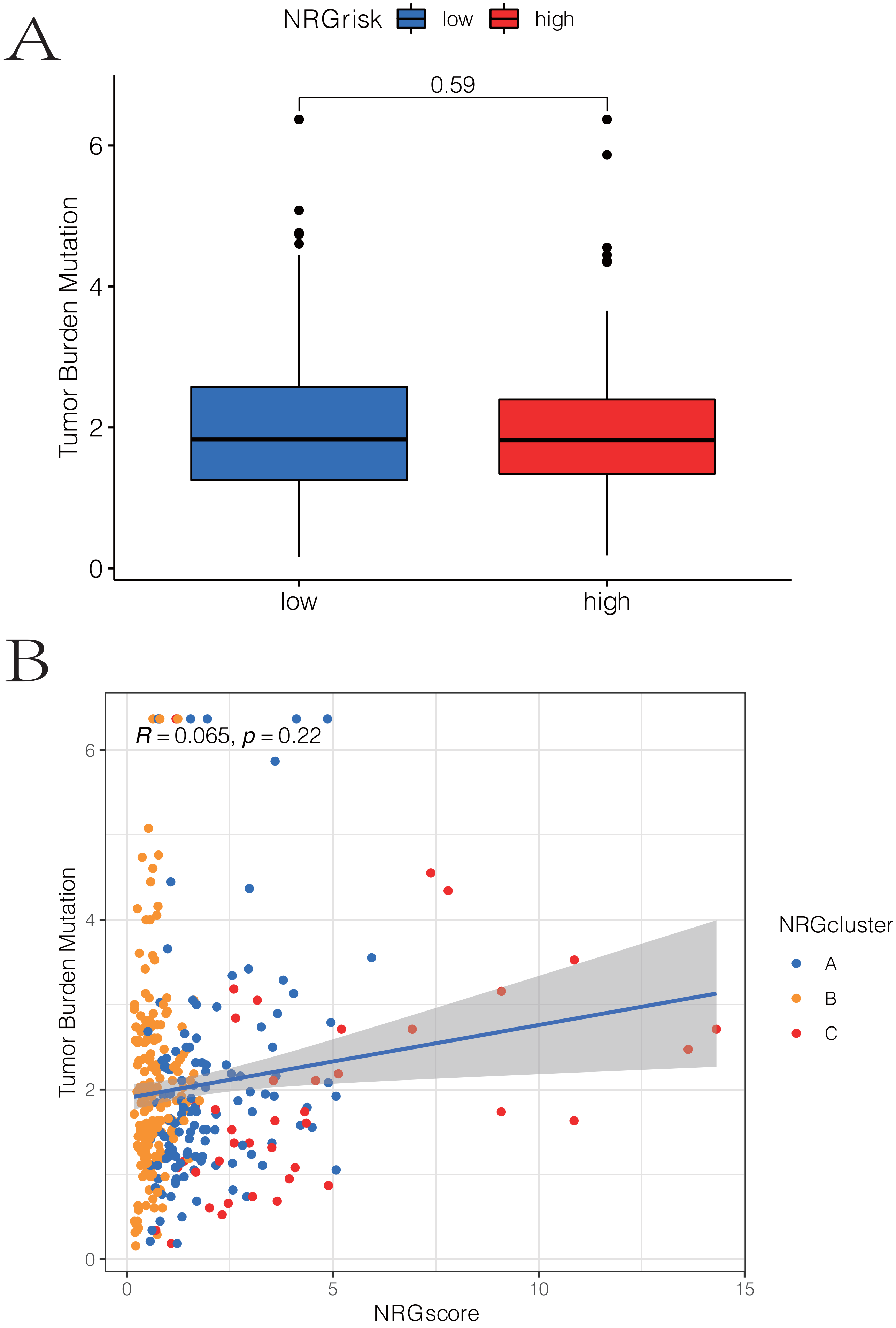

A
NRGrisk
low
high
0.59
Tumor Burden Mutation
6
4
2
0
low
high
B
R = 0.065, p = 0.22
Tumor Burden Mutation
6
4
2
0
0
5
10
15
NRGscore
NRGcluster
A
B
C
