# Supplementary material for: Computational Characterizing Necroptosis Reveals Implications for Immune Infiltration and Immunotherapy of Hepatocellular Carcinoma
Source: Front Oncol. 2022 Jul 7;12:933210. doi: 10.3389/fonc.2022.933210 (PMC9301124; doi:10.3389/fonc.2022.933210)
Supplement: Supplementary file 2 [file Table_1.docx]

Table S1: Necroptosis related genes identified from gene set or literature.

| **Genes** | **Sources** |
| --- | --- |
| FADD | GSEA necroptosis gene set |
| FAS | GSEA necroptosis gene set |
| FASLG | GSEA necroptosis gene set |
| MLKL | GSEA necroptosis gene set |
| RIPK1 | GSEA necroptosis gene set |
| RIPK3 | GSEA necroptosis gene set |
| TLR3 | GSEA necroptosis gene set |
| TNF | GSEA necroptosis gene set |
| TSC1 | PMID: 31961824 |
| TRIM11 | PMID: 31961824 |
| CASP8 | PMID: 31748744 |
| ZBP1 | PMID: 31076724 |
| MAPK8 | PMID: 31706322 |
| IPMK | PMID: 29883610 |
| ITPK1 | PMID: 29883610 |
| SIRT3 | PMID: 31894331 |
| MYC | PMID: 32753382 |
| TNFRSF1A | PMID: 27049944 |
| TNFSF10 | PMID: 27049944 |
| TNFRSF1B | PMID: 26993379 |
| TRAF2 | PMID: 26993379 |
| PANX1 | PMID: 31410978 |
| OTULIN | PMID: 29950720 |
| CYLD | PMID: 28362430 |
| USP22 | PMID: 33369872 |
| MAP3K7 | PMID: 27219062 |
| SQSTM1 | PMID: 27219062 |
| STAT3 | PMID: 32100392 |
| DIABLO | PMID: 27194728 |
| DNMT1 | PMID: 32554751 |
| CFLAR | PMID: 30518925 |
| BRAF | PMID: 30157175 |
| AXL | PMID: 30157175 |
| ID1 | PMID: 32004572 |
| CDKN2A | PMID: 28811972 |
| HSPA4 | PMID: 32156734 |
| BCL2 | PMID: 33239070 |
| STUB1 | PMID: 29686306 |
| FLT3 | PMID: 30828789 |
| HAT1 | PMID: 29535128 |
| SIRT2 | PMID: 29535128 |
| SIRT1 | PMID: 29535128 |
| PLK1 | PMID: 22890325 |
| MPG | PMID: 30755477 |
| BACH2 | PMID: 31918262 |
| GATA3 | PMID: 31918262 |
| MYCN | PMID: 26633716 |
| ALK | PMID: 26633716 |
| ATRX | PMID: 26633716 |
| TERT | PMID: 26633716 |
| SLC39A7 | PMID: 30237509 |
| SPATA2 | PMID: 27545878 |
| RNF31 | PMID: 27545878 |
| IDH1 | PMID: 28564603 |
| IDH2 | PMID: 28564603 |
| KLF9 | PMID: 30348136 |
| HDAC9 | PMID: 30348136 |
| HSP90AA1 | PMID: 23147571 |
| LEF1 | PMID: 22157808 |
| BNIP3 | PMID: 20963496 |
| CD40 | PMID: 26313915 |
| BCL2L11 | PMID: 24561519 |
| EGFR | PMID: 25688715 |
| DDX58 | PMID: 33852834 |
| TARDBP | PMID: 33852834 |
| APP | PMID: 34105277 |
| TNFRSF21 | PMID: 34105277 |
