# Supplementary material for: Computational Characterizing Necroptosis Reveals Implications for Immune Infiltration and Immunotherapy of Hepatocellular Carcinoma
Source: Front Oncol. 2022 Jul 7;12:933210. doi: 10.3389/fonc.2022.933210 (PMC9301124; doi:10.3389/fonc.2022.933210)
Supplement: Supplementary file 3 [file Table_2.docx]

Table S2: Basic clinical information of HCC patients from two cohorts.

|  | TCGA-LIHC | IGCG-LIRI |
| --- | --- | --- |
| Sample size | 370 | 229 |
| Age | 59.2 (13.4) | 67.2 (10.1) |
| Gender: |  |  |
| FEMALE | 118 (31.9%) | 60 (26.2%) |
| MALE | 252 (68.1%) | 169 (73.8%) |
| Grade: |  |  |
| G1 | 54 (14.6%) |  |
| G2 | 180 (48.6%) |  |
| G3 | 123 (33.2%) |  |
| G4 | 13 (3.51%) |  |
| Stage: |  |  |
| Stage I | 173 (46.8%) | 35 (15.3%) |
| Stage II | 85 (23.0%) | 104 (45.4%) |
| Stage III | 86 (23.2%) | 71 (31.0%) |
| Stage IV | 5 (1.35%) | 19 (8.30%) |
| unknow | 21 (5.68%) |  |
| T: |  |  |
| T1 | 184 (49.7%) |  |
| T2 | 92 (24.9%) |  |
| T3 | 81 (21.9%) |  |
| T4 | 13 (3.51%) |  |
| M: |  |  |
| M0 | 270 (73.0%) |  |
| M1 | 4 (1.08%) |  |
| unknow | 96 (25.9%) |  |
| N: |  |  |
| N0 | 256 (69.2%) |  |
| N1 | 4 (1.08%) |  |
| unknow | 110 (29.7%) |  |
