# Supplementary material for: Computational Characterizing Necroptosis Reveals Implications for Immune Infiltration and Immunotherapy of Hepatocellular Carcinoma
Source: Front Oncol. 2022 Jul 7;12:933210. doi: 10.3389/fonc.2022.933210 (PMC9301124; doi:10.3389/fonc.2022.933210)
Supplement: Supplementary file 7 [file Table_6.docx]

| Immune check point | R correlation | P value |
| --- | --- | --- |
| PD1 | 0.244252448 | 1.38E-09 |
| PD- L1 | 0.200350625 | 7.68E-07 |
| PD- L2 | 0.150438393 | 0.000219662 |
| CTLA4 | 0.272464335 | 1.18E-11 |
| TIM- 3 | 0.28400541 | 1.41E-12 |
| IDO1 | 0.143847584 | 0.000413017 |
| LAG3 | 0.209093839 | 2.42E-07 |
| TIGIT | 0.243150465 | 1.65E-09 |

Table S6: Relationship between NRGscore and expression of genes for immune check point.
