# Supplementary material for: Computational Characterizing Necroptosis Reveals Implications for Immune Infiltration and Immunotherapy of Hepatocellular Carcinoma
Source: Front Oncol. 2022 Jul 7;12:933210. doi: 10.3389/fonc.2022.933210 (PMC9301124; doi:10.3389/fonc.2022.933210)
Supplement: Supplementary file 8 [file Table_7.docx]

Table S7: Relationship between NRGscore and IPS score in HCC patients.

| IPS score | NRGScore | | χ2 value | P value |
| --- | --- | --- | --- | --- |
|  | low | high |  |  |
| 5 | 3 | 0 | 18.551 | 0.002329 |
| 6 | 17 | 13 |  |  |
| 7 | 99 | 58 |  |  |
| 8 | 114 | 116 |  |  |
| 9 | 61 | 83 |  |  |
| 10 | 14 | 21 |  |  |
